# Bis(*N*-benzyl-*N*-isopropyl­dithio­carbamato-κ*S*)di-*n*-butyl­tin(IV)

**DOI:** 10.1107/S1600536809015116

**Published:** 2009-04-30

**Authors:** Ibrahim Baba, Nor Syaidatul Akmal, Normah Awang, Seik Weng Ng

**Affiliations:** a School of Chemical Sciences and Food Technology, Universiti Kebangsaan Malaysia, 43600 Bangi, Selangor Darul Ehsan, Malaysia; b Department of Chemistry, University of Malaya, 50603 Kuala Lumpur, Malaysia

## Abstract

The Sn atom in the title compound, [Sn(C_4_H_9_)_2_(C_11_H_14_NS_2_)_2_], exists in a tetra­hedral C_2_S_2_Sn coordination geometry. The geometry is distorted towards skew-trapezoidal-bipyramidal owing to the proximity of the double-bonded S atoms. The C_2_Sn angles range from 129.0 (2) to 136.9 (2)°, the covalent Sn—S lengths from 2.529 (1) to 2.544 (1) Å, and the dative Sn←S lengths from 2.831 (1) to 3.042 (1) Å in the five independent mol­ecules comprising the asymmetric unit. Two of the butyl groups were modelled over two positions of equal occupancy. All butyl groups were refined with distance restraints.

## Related literature

For other *di*-n-butyl­tin dithio­carbamates, see: Farina *et al.* (2000 ▶); Lokaj *et al.* (1986 ▶); Menezes *et al.* (2005 ▶); Vrábel *et al.* (1992*a* ▶,*b* ▶); Vrábel & Kellö (1993 ▶); Zia-ur-Rehman *et al.* (2006 ▶). For a review of the applications and structures of tin dithio­carbamates, see: Tiekink (2008 ▶).
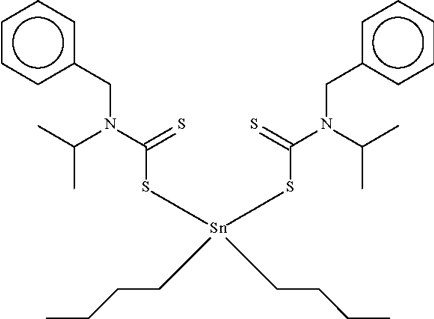


## Experimental

### 

#### Crystal data

[Sn(C_4_H_9_)_2_(C_11_H_14_NS_2_)_2_]
*M* *_r_* = 681.62
Triclinic, 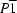

*a* = 17.7745 (2) Å
*b* = 19.5463 (3) Å
*c* = 26.2062 (4) Å
α = 102.5254 (7)°
β = 95.1492 (7)°
γ = 110.2569 (8)°
*V* = 8202.1 (2) Å^3^
*Z* = 10
Mo *K*α radiation
μ = 1.06 mm^−1^
*T* = 123 K
0.30 × 0.30 × 0.10 mm

#### Data collection

Bruker SMART APEX diffractometer
Absorption correction: multi-scan (*SADABS*; Sheldrick, 1996 ▶) *T*_min_ = 0.743, *T*_max_ = 0.902
67722 measured reflections
36923 independent reflections
23409 reflections with *I* > 2σ(*I*)
*R*_int_ = 0.028

#### Refinement

*R*[*F* ^2^ > 2σ(*F* ^2^)] = 0.046
*wR*(*F* ^2^) = 0.139
*S* = 1.15
36923 reflections
1684 parameters
96 restraints
H-atom parameters constrained
Δρ_max_ = 2.53 e Å^−3^
Δρ_min_ = −1.45 e Å^−3^

### 

Data collection: *APEX2* (Bruker, 2008 ▶); cell refinement: *SAINT* (Bruker, 2008 ▶); data reduction: *SAINT*; program(s) used to solve structure: *SHELXS97* (Sheldrick, 2008 ▶); program(s) used to refine structure: *SHELXL97* (Sheldrick, 2008 ▶); molecular graphics: *X-SEED* (Barbour, 2001 ▶); software used to prepare material for publication: *publCIF* (Westrip, 2009 ▶).

## Supplementary Material

Crystal structure: contains datablocks global, I. DOI: 10.1107/S1600536809015116/tk2429sup1.cif

Structure factors: contains datablocks I. DOI: 10.1107/S1600536809015116/tk2429Isup2.hkl

Additional supplementary materials:  crystallographic information; 3D view; checkCIF report

## Figures and Tables

**Fig. 1.**
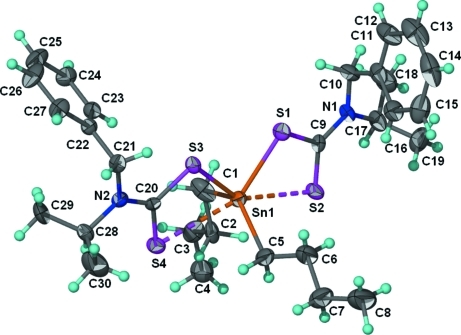
Thermal ellipsoid plot (Barbour, 2001) of one of the five independent molecules of dibutyldi(N- benzyl-N-isopropyldithiocarbamato)tin at the 70% probability level. Hydrogen atoms are drawn as spheres of arbitrary radius. The disorder is not shown.

## supplementary crystallographic information

### Experimental

Carbon disulfide (4 ml, 0.06 mol) was added to benzylisopropylamine (8 ml, 0.06 mol) in ethanol (50 ml) at 277 K. Dibutyltin dichloride (9.1 g, 0.03 mol) dissolved in ethanol (50 ml) was added. The white solid that precipitated was collected and recrystallized from an ethanol-chloroform mixture.

### Refinement

Somewhat tight distances restraints (1,2-related carbon-carbon distances were restrained to 1.540±0.005 Å and 1,3-related ones to 2.510±0.010 Å) were imposed on all butyl chains. Two of the chains are disordered over two positions; for these, the temperature factors of the primed atoms were restrained to those of the unprimed ones; their anisotropic displacement parameters were restrained to be nearly isotropic. The occupancy was arbitrarily fixed as 50:50.

Carbon-bound H-atoms were placed in calculated positions (C—H 0.95 to 0.99 Å) and were included in the refinement in the riding model approximation, with *U*(H) set to 1.2 to 1.5*U*(C). The final difference Fourier map had a large peak/deep hole in the vicinity of the Sn3 atom.

### Crystal data

**Table:**

[Sn(C_4_H_9_)_2_(C_11_H_14_NS_2_)_2_]	*Z* = 10
*M**_r_* = 681.62	*F*(000) = 3540
Triclinic, *P*1	*D*_x_ = 1.380 Mg m^−^^3^
Hall symbol: -P 1	Mo *K*α radiation, λ = 0.71073 Å
*a* = 17.7745 (2) Å	Cell parameters from 9858 reflections
*b* = 19.5463 (3) Å	θ = 2.2–28.3°
*c* = 26.2062 (4) Å	µ = 1.06 mm^−^^1^
α = 102.5254 (7)°	*T* = 123 K
β = 95.1492 (7)°	Block, colorless
γ = 110.2569 (8)°	0.30 × 0.30 × 0.10 mm
*V* = 8202.1 (2) Å^3^	

### Data collection

**Table:**

Bruker SMART APEX diffractometer	36923 independent reflections
Radiation source: fine-focus sealed tube	23409 reflections with *I* > 2σ(*I*)
graphite	*R*_int_ = 0.028
ω scans	θ_max_ = 27.5°, θ_min_ = 0.8°
Absorption correction: multi-scan (*SADABS*; Sheldrick, 1996)	*h* = −23→23
*T*_min_ = 0.743, *T*_max_ = 0.902	*k* = −24→25
67722 measured reflections	*l* = −32→34

### Refinement

**Table:**

Refinement on *F*^2^	Primary atom site location: structure-invariant direct methods
Least-squares matrix: full	Secondary atom site location: difference Fourier map
*R*[*F*^2^ > 2σ(*F*^2^)] = 0.046	Hydrogen site location: inferred from neighbouring sites
*wR*(*F*^2^) = 0.139	H-atom parameters constrained
*S* = 1.15	*w* = 1/[σ^2^(*F*_o_^2^) + (0.05*P*)^2^ + 5*P*] where *P* = (*F*_o_^2^ + 2*F*_c_^2^)/3
36923 reflections	(Δ/σ)_max_ = 0.001
1684 parameters	Δρ_max_ = 2.53 e Å^−^^3^
96 restraints	Δρ_min_ = −1.45 e Å^−^^3^

### Fractional atomic coordinates and isotropic or equivalent isotropic displacement parameters (Å^2^)

**Table:**

	*x*	*y*	*z*	*U*_iso_*/*U*_eq_	Occ. (<1)
Sn1	0.232256 (17)	0.439810 (18)	1.019238 (11)	0.02370 (8)	
Sn2	0.175644 (17)	0.150825 (18)	0.783916 (12)	0.02621 (8)	
Sn3	0.639428 (17)	0.036822 (17)	0.818119 (11)	0.02389 (8)	
Sn4	0.571833 (17)	−0.249780 (17)	0.598326 (12)	0.02604 (8)	
Sn5	0.031055 (17)	−0.358358 (17)	0.608830 (11)	0.02252 (8)	
S1	0.12379 (6)	0.47805 (6)	0.97841 (4)	0.0258 (2)	
S2	0.13011 (6)	0.33223 (7)	0.92277 (4)	0.0275 (3)	
S3	0.26314 (6)	0.56663 (6)	1.08468 (4)	0.0231 (2)	
S4	0.36647 (6)	0.48533 (6)	1.11426 (4)	0.0258 (2)	
S5	0.28552 (7)	0.11337 (6)	0.82395 (4)	0.0282 (3)	
S6	0.27648 (6)	0.25794 (6)	0.88035 (4)	0.0264 (3)	
S7	0.14437 (6)	0.02626 (6)	0.71619 (4)	0.0263 (2)	
S8	0.04118 (7)	0.10898 (7)	0.68910 (5)	0.0308 (3)	
S9	0.52819 (7)	0.07056 (7)	0.77536 (4)	0.0284 (3)	
S10	0.54200 (6)	−0.07283 (7)	0.72210 (4)	0.0273 (3)	
S11	0.66700 (7)	0.16165 (6)	0.88444 (4)	0.0272 (2)	
S12	0.77308 (7)	0.08198 (7)	0.91306 (5)	0.0329 (3)	
S13	0.68694 (6)	−0.28802 (6)	0.63039 (4)	0.0242 (2)	
S14	0.68046 (7)	−0.14425 (6)	0.69001 (4)	0.0264 (2)	
S15	0.53479 (7)	−0.37098 (7)	0.52521 (4)	0.0308 (3)	
S16	0.42879 (7)	−0.28494 (8)	0.51283 (6)	0.0480 (4)	
S17	−0.08574 (6)	−0.32517 (6)	0.57251 (4)	0.0233 (2)	
S18	−0.06705 (6)	−0.46318 (6)	0.51304 (4)	0.0232 (2)	
S19	0.05551 (7)	−0.24046 (7)	0.68313 (4)	0.0293 (3)	
S20	0.17120 (6)	−0.31553 (7)	0.69985 (4)	0.0278 (3)	
N1	0.0105 (2)	0.3789 (2)	0.89688 (13)	0.0221 (8)	
N2	0.37929 (19)	0.6216 (2)	1.17057 (13)	0.0219 (8)	
N3	0.39919 (19)	0.21452 (19)	0.90461 (13)	0.0210 (8)	
N4	0.01806 (19)	−0.0318 (2)	0.63640 (13)	0.0236 (8)	
N5	0.4276 (2)	−0.0265 (2)	0.68725 (14)	0.0280 (8)	
N6	0.7859 (2)	0.2184 (2)	0.96880 (13)	0.0261 (8)	
N7	0.8023 (2)	−0.19025 (19)	0.71167 (14)	0.0249 (8)	
N8	0.4064 (2)	−0.4179 (2)	0.44738 (14)	0.0302 (9)	
N9	−0.18603 (19)	−0.4187 (2)	0.48321 (13)	0.0222 (8)	
N10	0.1859 (2)	−0.1811 (2)	0.76023 (14)	0.0270 (8)	
C1	0.1753 (3)	0.3562 (2)	1.0593 (2)	0.0382 (12)	
H1A	0.1903	0.3770	1.0983	0.046*	0.50
H1B	0.1152	0.3358	1.0488	0.046*	0.50
H1C	0.1733	0.3851	1.0948	0.046*	0.50
H1D	0.1181	0.3308	1.0403	0.046*	0.50
C2	0.2103 (5)	0.2953 (4)	1.0399 (3)	0.0259 (14)	0.50
H2A	0.2007	0.2808	1.0005	0.031*	0.50
H2B	0.2699	0.3166	1.0528	0.031*	0.50
C3	0.1721 (5)	0.2261 (4)	1.0591 (4)	0.0349 (16)	0.50
H3A	0.1832	0.2414	1.0984	0.042*	0.50
H3B	0.1123	0.2071	1.0474	0.042*	0.50
C4	0.2001 (10)	0.1625 (6)	1.0405 (5)	0.040 (2)	0.50
H4A	0.1526	0.1152	1.0264	0.060*	0.50
H4B	0.2337	0.1581	1.0705	0.060*	0.50
H4C	0.2323	0.1726	1.0126	0.060*	0.50
C2'	0.2005 (5)	0.2940 (4)	1.0690 (3)	0.0259 (14)	0.50
H2C	0.1718	0.2727	1.0961	0.031*	0.50
H2D	0.2598	0.3141	1.0826	0.031*	0.50
C3'	0.1797 (6)	0.2330 (4)	1.0179 (3)	0.0349 (16)	0.50
H3C	0.1210	0.2168	1.0033	0.042*	0.50
H3D	0.2110	0.2546	0.9920	0.042*	0.50
C4'	0.1967 (10)	0.1647 (6)	1.0232 (5)	0.040 (2)	0.50
H4D	0.1451	0.1216	1.0177	0.060*	0.50
H4E	0.2272	0.1750	1.0589	0.060*	0.50
H4F	0.2289	0.1529	0.9967	0.060*	0.50
C5	0.3345 (2)	0.4515 (3)	0.97928 (15)	0.0312 (11)	
H5A	0.3842	0.4860	1.0053	0.037*	
H5B	0.3390	0.4014	0.9695	0.037*	
C6	0.3358 (3)	0.4798 (3)	0.93039 (17)	0.0459 (14)	
H6A	0.2874	0.4452	0.9033	0.055*	
H6B	0.3322	0.5303	0.9394	0.055*	
C7	0.4112 (3)	0.4854 (3)	0.90716 (17)	0.0412 (13)	
H7A	0.4591	0.5182	0.9352	0.049*	
H7B	0.4135	0.4344	0.8979	0.049*	
C8	0.4201 (4)	0.5152 (5)	0.8592 (2)	0.087 (3)	
H8A	0.4711	0.5155	0.8477	0.130*	
H8B	0.4208	0.5668	0.8679	0.130*	
H8C	0.3741	0.4827	0.8305	0.130*	
C9	0.0811 (2)	0.3927 (2)	0.92808 (15)	0.0176 (8)	
C10	−0.0271 (2)	0.4356 (3)	0.90241 (18)	0.0285 (10)	
H10A	−0.0161	0.4631	0.9404	0.034*	
H10B	−0.0869	0.4089	0.8915	0.034*	
C11	0.0017 (3)	0.4929 (2)	0.87057 (16)	0.0269 (10)	
C12	−0.0439 (3)	0.5376 (3)	0.8654 (2)	0.0406 (13)	
H12	−0.0907	0.5313	0.8816	0.049*	
C13	−0.0212 (3)	0.5907 (3)	0.8369 (2)	0.0503 (15)	
H13	−0.0527	0.6205	0.8334	0.060*	
C14	0.0469 (4)	0.6011 (3)	0.8135 (2)	0.0497 (15)	
H14	0.0620	0.6378	0.7938	0.060*	
C15	0.0928 (3)	0.5582 (3)	0.81852 (19)	0.0425 (13)	
H15	0.1396	0.5651	0.8023	0.051*	
C16	0.0706 (3)	0.5046 (3)	0.84753 (18)	0.0321 (11)	
H16	0.1032	0.4758	0.8515	0.038*	
C17	−0.0297 (3)	0.3055 (3)	0.85548 (17)	0.0295 (10)	
H17	−0.0103	0.2679	0.8670	0.035*	
C18	−0.1227 (3)	0.2748 (3)	0.84989 (19)	0.0400 (12)	
H18A	−0.1376	0.2716	0.8846	0.060*	
H18B	−0.1459	0.2244	0.8249	0.060*	
H18C	−0.1440	0.3089	0.8366	0.060*	
C19	−0.0048 (3)	0.3113 (3)	0.80264 (18)	0.0421 (13)	
H19A	0.0548	0.3305	0.8070	0.063*	
H19B	−0.0251	0.3460	0.7893	0.063*	
H19C	−0.0279	0.2612	0.7772	0.063*	
C20	0.3412 (2)	0.5626 (2)	1.12744 (15)	0.0180 (8)	
C21	0.3577 (2)	0.6888 (2)	1.18152 (16)	0.0224 (9)	
H21A	0.3319	0.6927	1.1477	0.027*	
H21B	0.4083	0.7343	1.1954	0.027*	
C22	0.3001 (2)	0.6880 (2)	1.22118 (15)	0.0202 (9)	
C23	0.2312 (2)	0.6243 (2)	1.21723 (16)	0.0231 (9)	
H23	0.2196	0.5799	1.1894	0.028*	
C24	0.1792 (3)	0.6250 (3)	1.25352 (17)	0.0290 (10)	
H24	0.1320	0.5812	1.2504	0.035*	
C25	0.1961 (3)	0.6892 (3)	1.29407 (18)	0.0360 (12)	
H25	0.1600	0.6902	1.3186	0.043*	
C26	0.2656 (3)	0.7521 (3)	1.29898 (18)	0.0385 (12)	
H26	0.2782	0.7958	1.3277	0.046*	
C27	0.3167 (3)	0.7520 (3)	1.26246 (17)	0.0291 (10)	
H27	0.3636	0.7960	1.2656	0.035*	
C28	0.4493 (2)	0.6239 (3)	1.20749 (16)	0.0278 (10)	
H28	0.4458	0.5706	1.2023	0.033*	
C29	0.4449 (3)	0.6526 (4)	1.26458 (18)	0.0579 (18)	
H29A	0.3918	0.6230	1.2717	0.087*	
H29B	0.4515	0.7059	1.2718	0.087*	
H29C	0.4884	0.6478	1.2875	0.087*	
C30	0.5282 (3)	0.6670 (4)	1.1931 (2)	0.0596 (18)	
H30A	0.5282	0.6454	1.1556	0.089*	
H30B	0.5734	0.6639	1.2155	0.089*	
H30C	0.5346	0.7201	1.1986	0.089*	
C31	0.0746 (3)	0.1377 (3)	0.82472 (16)	0.0340 (11)	
H31A	0.0739	0.1886	0.8393	0.041*	
H31B	0.0240	0.1085	0.7981	0.041*	
C32	0.0708 (2)	0.0998 (3)	0.86955 (16)	0.0379 (12)	
H32A	0.1212	0.1274	0.8966	0.046*	
H32B	0.0674	0.0473	0.8555	0.046*	
C33	−0.0026 (3)	0.0986 (3)	0.89513 (19)	0.0471 (14)	
H33A	0.0036	0.1514	0.9113	0.056*	
H33B	−0.0519	0.0756	0.8670	0.056*	
C34	−0.0157 (3)	0.0561 (3)	0.9371 (2)	0.0588 (17)	
H34A	−0.0641	0.0582	0.9515	0.088*	
H34B	0.0321	0.0792	0.9657	0.088*	
H34C	−0.0236	0.0033	0.9213	0.088*	
C35	0.2309 (3)	0.2365 (2)	0.74597 (19)	0.0369 (12)	
H35A	0.2908	0.2578	0.7579	0.044*	
H35B	0.2192	0.2148	0.7070	0.044*	
C36	0.1971 (3)	0.2984 (3)	0.7601 (2)	0.0528 (15)	
H36A	0.1373	0.2761	0.7474	0.063*	
H36B	0.2067	0.3174	0.7993	0.063*	
C37	0.2324 (4)	0.3634 (3)	0.7376 (3)	0.075 (2)	
H37A	0.2156	0.3461	0.6984	0.090*	
H37B	0.2926	0.3813	0.7457	0.090*	
C38	0.2067 (4)	0.4285 (3)	0.7587 (3)	0.0600 (17)	
H38A	0.2328	0.4700	0.7430	0.090*	
H38B	0.2234	0.4461	0.7975	0.090*	
H38C	0.1474	0.4118	0.7494	0.090*	
C39	0.3278 (2)	0.1983 (2)	0.87374 (15)	0.0169 (8)	
C40	0.4393 (2)	0.1594 (3)	0.89916 (18)	0.0277 (10)	
H40A	0.4984	0.1872	0.9131	0.033*	
H40B	0.4330	0.1352	0.8609	0.033*	
C41	0.4080 (2)	0.0982 (2)	0.92699 (16)	0.0242 (10)	
C42	0.4479 (3)	0.0478 (3)	0.92573 (19)	0.0355 (12)	
H42	0.4931	0.0533	0.9080	0.043*	
C43	0.4217 (3)	−0.0101 (3)	0.9501 (2)	0.0491 (15)	
H43	0.4482	−0.0450	0.9482	0.059*	
C44	0.3580 (3)	−0.0180 (3)	0.9770 (2)	0.0463 (14)	
H44	0.3414	−0.0573	0.9944	0.056*	
C45	0.3180 (3)	0.0312 (3)	0.9789 (2)	0.0426 (13)	
H45	0.2735	0.0257	0.9973	0.051*	
C46	0.3430 (3)	0.0892 (3)	0.95364 (19)	0.0340 (11)	
H46	0.3150	0.1229	0.9548	0.041*	
C47	0.4395 (2)	0.2887 (2)	0.94499 (16)	0.0256 (10)	
H47	0.4174	0.3247	0.9336	0.031*	
C48	0.4180 (3)	0.2841 (3)	0.99917 (18)	0.0395 (12)	
H48A	0.3586	0.2632	0.9961	0.059*	
H48B	0.4404	0.3348	1.0238	0.059*	
H48C	0.4412	0.2512	1.0126	0.059*	
C49	0.5320 (2)	0.3218 (3)	0.94771 (18)	0.0326 (11)	
H49A	0.5438	0.3239	0.9121	0.049*	
H49B	0.5564	0.2896	0.9610	0.049*	
H49C	0.5549	0.3729	0.9718	0.049*	
C50	0.0624 (2)	0.0298 (2)	0.67701 (16)	0.0203 (9)	
C51	0.0444 (2)	−0.0955 (2)	0.62089 (16)	0.0241 (9)	
H51A	−0.0041	−0.1433	0.6106	0.029*	
H51B	0.0794	−0.0968	0.6520	0.029*	
C52	0.0911 (2)	−0.0914 (2)	0.57531 (16)	0.0233 (9)	
C53	0.1626 (3)	−0.0300 (3)	0.57913 (18)	0.0302 (10)	
H53	0.1822	0.0105	0.6106	0.036*	
C54	0.2052 (3)	−0.0270 (3)	0.53796 (19)	0.0346 (11)	
H54	0.2541	0.0151	0.5411	0.041*	
C55	0.1766 (3)	−0.0858 (3)	0.4917 (2)	0.0419 (13)	
H55	0.2062	−0.0843	0.4632	0.050*	
C56	0.1053 (3)	−0.1461 (3)	0.4872 (2)	0.0458 (14)	
H56	0.0853	−0.1857	0.4552	0.055*	
C57	0.0623 (3)	−0.1499 (3)	0.52883 (18)	0.0361 (12)	
H57	0.0135	−0.1921	0.5256	0.043*	
C58	−0.0575 (2)	−0.0350 (3)	0.60476 (18)	0.0344 (12)	
H58	−0.0766	0.0019	0.6271	0.041*	
C59	−0.0408 (3)	−0.0100 (3)	0.5559 (2)	0.0471 (14)	
H59A	0.0028	0.0403	0.5654	0.071*	
H59B	−0.0903	−0.0078	0.5378	0.071*	
H59C	−0.0240	−0.0460	0.5321	0.071*	
C60	−0.1255 (3)	−0.1117 (3)	0.5922 (2)	0.0563 (17)	
H60A	−0.1738	−0.1109	0.5715	0.084*	
H60B	−0.1388	−0.1241	0.6254	0.084*	
H60C	−0.1081	−0.1499	0.5716	0.084*	
C61	0.5850 (2)	−0.0514 (2)	0.85512 (17)	0.0279 (10)	
H61A	0.5931	−0.0296	0.8939	0.033*	
H61B	0.5257	−0.0753	0.8412	0.033*	
C62	0.6226 (2)	−0.1104 (2)	0.84437 (17)	0.0301 (10)	
H62A	0.6172	−0.1295	0.8055	0.036*	
H62B	0.6815	−0.0862	0.8596	0.036*	
C63	0.5850 (3)	−0.1763 (2)	0.8668 (2)	0.0445 (13)	
H63A	0.5252	−0.1968	0.8549	0.053*	
H63B	0.5961	−0.1581	0.9061	0.053*	
C64	0.6158 (3)	−0.2392 (3)	0.8509 (2)	0.0492 (14)	
H64A	0.5889	−0.2800	0.8669	0.074*	
H64B	0.6749	−0.2198	0.8632	0.074*	
H64C	0.6037	−0.2587	0.8121	0.074*	
C65	0.7446 (3)	0.0529 (3)	0.78004 (16)	0.0375 (12)	
H65A	0.7915	0.0941	0.8050	0.045*	
H65B	0.7564	0.0063	0.7754	0.045*	
C66	0.7418 (3)	0.0712 (3)	0.72747 (18)	0.0474 (14)	
H66A	0.7298	0.1177	0.7311	0.057*	
H66B	0.6970	0.0294	0.7013	0.057*	
C67	0.8207 (3)	0.0827 (3)	0.70708 (19)	0.0529 (15)	
H67A	0.8645	0.1268	0.7322	0.063*	
H67B	0.8346	0.0378	0.7066	0.063*	
C68	0.8193 (4)	0.0954 (5)	0.6528 (2)	0.084 (2)	
H68A	0.8726	0.1024	0.6425	0.125*	
H68B	0.8071	0.1406	0.6529	0.125*	
H68C	0.7773	0.0514	0.6272	0.125*	
C69	0.4934 (2)	−0.0124 (2)	0.72396 (15)	0.0202 (9)	
C70	0.3804 (3)	0.0228 (3)	0.69312 (19)	0.0350 (11)	
H70A	0.3837	0.0440	0.7315	0.042*	
H70B	0.3225	−0.0089	0.6778	0.042*	
C71	0.4067 (3)	0.0873 (3)	0.66764 (17)	0.0313 (11)	
C72	0.3584 (3)	0.1296 (3)	0.6668 (2)	0.0421 (13)	
H72	0.3115	0.1187	0.6831	0.050*	
C73	0.3784 (3)	0.1875 (3)	0.6425 (2)	0.0524 (16)	
H73	0.3454	0.2167	0.6425	0.063*	
C74	0.4450 (4)	0.2036 (3)	0.6180 (2)	0.0555 (17)	
H74	0.4570	0.2426	0.6003	0.067*	
C75	0.4950 (3)	0.1625 (3)	0.6193 (2)	0.0477 (14)	
H75	0.5418	0.1737	0.6029	0.057*	
C76	0.4756 (3)	0.1050 (3)	0.64475 (19)	0.0373 (12)	
H76	0.5102	0.0775	0.6465	0.045*	
C77	0.3970 (3)	−0.0965 (3)	0.64221 (18)	0.0320 (11)	
H77	0.4435	−0.1134	0.6373	0.038*	
C78	0.3687 (3)	−0.0837 (3)	0.59042 (19)	0.0479 (14)	
H78A	0.4127	−0.0428	0.5825	0.072*	
H78B	0.3210	−0.0698	0.5932	0.072*	
H78C	0.3541	−0.1302	0.5619	0.072*	
C79	0.3304 (3)	−0.1594 (3)	0.6568 (2)	0.0458 (13)	
H79A	0.3510	−0.1662	0.6905	0.069*	
H79B	0.3150	−0.2067	0.6288	0.069*	
H79C	0.2827	−0.1457	0.6603	0.069*	
C80	0.7481 (2)	0.1587 (2)	0.92652 (15)	0.0206 (9)	
C81	0.7632 (2)	0.2844 (2)	0.98009 (16)	0.0252 (10)	
H81A	0.7355	0.2873	0.9466	0.030*	
H81B	0.8135	0.3304	0.9930	0.030*	
C82	0.7079 (2)	0.2838 (2)	1.02081 (16)	0.0236 (9)	
C83	0.7263 (3)	0.3477 (3)	1.06246 (19)	0.0373 (12)	
H83	0.7734	0.3914	1.0651	0.045*	
C84	0.6770 (3)	0.3486 (3)	1.1001 (2)	0.0447 (13)	
H84	0.6908	0.3923	1.1289	0.054*	
C85	0.6075 (3)	0.2859 (3)	1.0959 (2)	0.0463 (14)	
H85	0.5725	0.2870	1.1212	0.056*	
C86	0.5890 (3)	0.2210 (3)	1.05468 (19)	0.0350 (11)	
H86	0.5418	0.1774	1.0520	0.042*	
C87	0.6395 (2)	0.2205 (3)	1.01777 (17)	0.0258 (10)	
H87	0.6272	0.1760	0.9899	0.031*	
C88	0.8572 (3)	0.2209 (3)	1.00522 (17)	0.0341 (12)	
H88	0.8511	0.1673	1.0023	0.041*	
C89	0.9346 (3)	0.2579 (4)	0.9872 (2)	0.0645 (19)	
H89A	0.9312	0.2323	0.9501	0.097*	
H89B	0.9801	0.2548	1.0094	0.097*	
H89C	0.9435	0.3112	0.9904	0.097*	
C90	0.8588 (3)	0.2559 (4)	1.0620 (2)	0.068 (2)	
H90A	0.8064	0.2302	1.0719	0.102*	
H90B	0.8685	0.3096	1.0671	0.102*	
H90C	0.9027	0.2507	1.0845	0.102*	
C91	0.6223 (2)	−0.1645 (2)	0.55824 (16)	0.0267 (10)	
H91A	0.6825	−0.1440	0.5685	0.032*	
H91B	0.6086	−0.1883	0.5195	0.032*	
C92	0.5935 (2)	−0.0999 (2)	0.56962 (16)	0.0260 (10)	
H92A	0.6093	−0.0744	0.6081	0.031*	
H92B	0.5332	−0.1203	0.5606	0.031*	
C93	0.6277 (3)	−0.0423 (2)	0.53913 (18)	0.0369 (12)	
H93A	0.6879	−0.0253	0.5457	0.044*	
H93B	0.6080	−0.0669	0.5007	0.044*	
C94	0.6047 (3)	0.0260 (3)	0.5536 (2)	0.0510 (15)	
H94A	0.6294	0.0612	0.5328	0.076*	
H94B	0.6246	0.0512	0.5916	0.076*	
H94C	0.5453	0.0100	0.5459	0.076*	
C95	0.4772 (3)	−0.2775 (3)	0.6439 (2)	0.062 (2)	
H95A	0.4698	−0.2296	0.6586	0.075*	0.50
H95B	0.4270	−0.3104	0.6178	0.075*	0.50
H95C	0.4551	−0.2369	0.6498	0.075*	0.50
H95D	0.4325	−0.3249	0.6232	0.075*	0.50
C96	0.4764 (6)	−0.3133 (8)	0.6880 (4)	0.071 (3)	0.50
H96A	0.5268	−0.2847	0.7149	0.085*	0.50
H96B	0.4732	−0.3659	0.6746	0.085*	0.50
C97	0.4018 (5)	−0.3128 (6)	0.7125 (4)	0.0449 (19)	0.50
H97A	0.4075	−0.2599	0.7277	0.054*	0.50
H97B	0.3524	−0.3374	0.6845	0.054*	0.50
C98	0.3925 (7)	−0.3535 (7)	0.7551 (4)	0.069 (3)	0.50
H98A	0.3393	−0.3608	0.7655	0.103*	0.50
H98B	0.4358	−0.3235	0.7859	0.103*	0.50
H98C	0.3963	−0.4028	0.7417	0.103*	0.50
C96'	0.5050 (5)	−0.2873 (8)	0.6971 (4)	0.071 (3)	0.50
H96C	0.5436	−0.3134	0.6918	0.085*	0.50
H96D	0.5364	−0.2361	0.7209	0.085*	0.50
C97'	0.4438 (5)	−0.3289 (5)	0.7268 (3)	0.0449 (19)	0.50
H97C	0.4107	−0.3795	0.7028	0.054*	0.50
H97D	0.4742	−0.3368	0.7571	0.054*	0.50
C98'	0.3861 (7)	−0.2938 (8)	0.7480 (5)	0.069 (3)	0.50
H98D	0.3706	−0.3098	0.7798	0.103*	0.50
H98E	0.3373	−0.3100	0.7209	0.103*	0.50
H98F	0.4129	−0.2385	0.7573	0.103*	0.50
C99	0.7298 (2)	−0.2053 (2)	0.68204 (15)	0.0211 (9)	
C100	0.8413 (2)	−0.2462 (2)	0.70375 (17)	0.0270 (10)	
H10C	0.9005	−0.2195	0.7178	0.032*	
H10D	0.8347	−0.2683	0.6651	0.032*	
C101	0.8087 (2)	−0.3097 (2)	0.72959 (16)	0.0241 (9)	
C102	0.8428 (3)	−0.3643 (3)	0.72254 (18)	0.0343 (11)	
H10E	0.8850	−0.3605	0.7022	0.041*	
C103	0.8159 (3)	−0.4240 (3)	0.7448 (2)	0.0427 (13)	
H10F	0.8394	−0.4613	0.7394	0.051*	
C104	0.7551 (3)	−0.4303 (3)	0.7751 (2)	0.0428 (13)	
H10G	0.7368	−0.4717	0.7903	0.051*	
C105	0.7217 (3)	−0.3758 (3)	0.7827 (2)	0.0394 (12)	
H10H	0.6808	−0.3789	0.8041	0.047*	
C106	0.7472 (3)	−0.3171 (3)	0.75957 (18)	0.0330 (11)	
H10I	0.7223	−0.2808	0.7642	0.040*	
C107	0.8446 (3)	−0.1171 (3)	0.75250 (18)	0.0363 (12)	
H10J	0.8189	−0.0814	0.7449	0.044*	
C108	0.8351 (4)	−0.1237 (3)	0.8068 (2)	0.0658 (19)	
H10K	0.7770	−0.1450	0.8085	0.099*	
H10L	0.8604	−0.0735	0.8320	0.099*	
H10M	0.8616	−0.1568	0.8161	0.099*	
C109	0.9354 (3)	−0.0818 (3)	0.7500 (2)	0.0630 (18)	
H10N	0.9415	−0.0774	0.7139	0.095*	
H10O	0.9632	−0.1141	0.7594	0.095*	
H10P	0.9597	−0.0315	0.7751	0.095*	
C110	0.4513 (3)	−0.3632 (3)	0.49128 (19)	0.0301 (10)	
C111	0.4308 (3)	−0.4810 (3)	0.42554 (18)	0.0356 (12)	
H11A	0.3814	−0.5279	0.4132	0.043*	
H11B	0.4656	−0.4874	0.4543	0.043*	
C112	0.4765 (3)	−0.4715 (3)	0.38000 (19)	0.0398 (13)	
C113	0.4468 (4)	−0.5250 (3)	0.3314 (2)	0.0530 (15)	
H11C	0.3965	−0.5664	0.3265	0.064*	
C114	0.4893 (4)	−0.5190 (4)	0.2903 (2)	0.0620 (18)	
H11D	0.4681	−0.5563	0.2572	0.074*	
C115	0.5613 (4)	−0.4603 (4)	0.2964 (2)	0.0570 (17)	
H11E	0.5907	−0.4569	0.2679	0.068*	
C116	0.5917 (3)	−0.4050 (3)	0.3449 (2)	0.0456 (14)	
H11F	0.6417	−0.3635	0.3494	0.055*	
C117	0.5492 (3)	−0.4109 (3)	0.38595 (19)	0.0393 (13)	
H11G	0.5698	−0.3731	0.4188	0.047*	
C118	0.3315 (3)	−0.4151 (3)	0.41884 (19)	0.0422 (13)	
H11H	0.3117	−0.3821	0.4444	0.051*	
C119	0.3501 (3)	−0.3803 (4)	0.3728 (2)	0.071 (2)	
H11I	0.3932	−0.3298	0.3860	0.107*	
H11J	0.3009	−0.3762	0.3560	0.107*	
H11K	0.3684	−0.4123	0.3467	0.107*	
C120	0.2643 (3)	−0.4936 (4)	0.4004 (2)	0.0635 (19)	
H12B	0.2536	−0.5145	0.4310	0.095*	
H12C	0.2815	−0.5267	0.3744	0.095*	
H12D	0.2145	−0.4901	0.3839	0.095*	
C121	0.1329 (3)	−0.3201 (3)	0.57042 (15)	0.0406 (13)	
H12E	0.1657	−0.2671	0.5905	0.049*	
H12V	0.1666	−0.3500	0.5756	0.049*	
C122	0.1232 (2)	−0.3215 (3)	0.51261 (15)	0.0331 (11)	
H12F	0.0993	−0.3747	0.4909	0.040*	
H12G	0.0848	−0.2969	0.5051	0.040*	
C123	0.2025 (3)	−0.2821 (3)	0.49689 (16)	0.0472 (14)	
H12H	0.2223	−0.2277	0.5157	0.057*	
H12I	0.2427	−0.3020	0.5096	0.057*	
C124	0.2013 (3)	−0.2889 (4)	0.43889 (17)	0.0603 (18)	
H12J	0.2558	−0.2601	0.4333	0.090*	
H12K	0.1623	−0.2688	0.4256	0.090*	
H12L	0.1850	−0.3422	0.4197	0.090*	
C125	−0.0164 (2)	−0.4480 (2)	0.64587 (16)	0.0237 (9)	
H12M	−0.0062	−0.4261	0.6848	0.028*	
H12N	−0.0762	−0.4723	0.6337	0.028*	
C126	0.0198 (2)	−0.5077 (2)	0.63444 (15)	0.0249 (9)	
H12O	0.0799	−0.4831	0.6448	0.030*	
H12P	0.0069	−0.5318	0.5957	0.030*	
C127	−0.0107 (3)	−0.5682 (2)	0.66324 (18)	0.0343 (11)	
H12Q	0.0082	−0.5451	0.7019	0.041*	
H12R	−0.0711	−0.5884	0.6565	0.041*	
C128	0.0174 (3)	−0.6329 (3)	0.6468 (2)	0.0457 (13)	
H12S	−0.0047	−0.6703	0.6666	0.069*	
H12T	0.0771	−0.6137	0.6544	0.069*	
H12U	−0.0018	−0.6567	0.6086	0.069*	
C129	−0.1197 (2)	−0.4052 (2)	0.51823 (14)	0.0144 (8)	
C130	−0.2356 (2)	−0.3721 (2)	0.49218 (17)	0.0250 (10)	
H13B	−0.2333	−0.3548	0.5309	0.030*	
H13C	−0.2930	−0.4042	0.4759	0.030*	
C131	−0.2102 (2)	−0.3034 (3)	0.47021 (16)	0.0248 (10)	
C132	−0.1437 (3)	−0.2830 (3)	0.44520 (18)	0.0316 (11)	
H13D	−0.1103	−0.3119	0.4423	0.038*	
C133	−0.1250 (3)	−0.2211 (3)	0.4244 (2)	0.0413 (13)	
H13E	−0.0798	−0.2082	0.4067	0.050*	
C134	−0.1730 (3)	−0.1779 (3)	0.42969 (19)	0.0408 (13)	
H13F	−0.1609	−0.1356	0.4152	0.049*	
C135	−0.2380 (3)	−0.1962 (3)	0.4558 (2)	0.0417 (13)	
H13G	−0.2698	−0.1657	0.4602	0.050*	
C136	−0.2570 (3)	−0.2591 (3)	0.47543 (17)	0.0308 (11)	
H13H	−0.3027	−0.2722	0.4928	0.037*	
C137	−0.2162 (3)	−0.4868 (3)	0.43653 (16)	0.0285 (10)	
H13I	−0.1682	−0.5001	0.4286	0.034*	
C138	−0.2512 (3)	−0.4723 (3)	0.38708 (18)	0.0416 (13)	
H13J	−0.2106	−0.4288	0.3793	0.062*	
H13K	−0.3002	−0.4615	0.3928	0.062*	
H13L	−0.2655	−0.5170	0.3571	0.062*	
C139	−0.2760 (3)	−0.5534 (3)	0.4505 (2)	0.0453 (13)	
H13M	−0.2511	−0.5611	0.4826	0.068*	
H13N	−0.2907	−0.5988	0.4210	0.068*	
H13O	−0.3252	−0.5433	0.4570	0.068*	
C140	0.1430 (2)	−0.2407 (2)	0.71770 (16)	0.0204 (9)	
C141	0.1560 (3)	−0.1208 (3)	0.77940 (18)	0.0332 (11)	
H14B	0.1195	−0.1186	0.7495	0.040*	
H14C	0.2032	−0.0719	0.7906	0.040*	
C142	0.1109 (3)	−0.1293 (3)	0.82486 (18)	0.0354 (12)	
C143	0.0414 (3)	−0.1924 (3)	0.82053 (19)	0.0369 (12)	
H14D	0.0236	−0.2328	0.7890	0.044*	
C144	−0.0024 (4)	−0.1973 (4)	0.8615 (2)	0.0524 (15)	
H14E	−0.0504	−0.2405	0.8580	0.063*	
C145	0.0245 (5)	−0.1387 (5)	0.9076 (3)	0.078 (2)	
H14F	−0.0055	−0.1417	0.9359	0.094*	
C146	0.0942 (5)	−0.0763 (5)	0.9129 (3)	0.080 (2)	
H14G	0.1129	−0.0369	0.9449	0.096*	
C147	0.1369 (4)	−0.0711 (4)	0.8715 (2)	0.0581 (17)	
H14H	0.1843	−0.0274	0.8749	0.070*	
C148	0.2647 (2)	−0.1753 (3)	0.78910 (18)	0.0339 (11)	
H14I	0.2850	−0.2096	0.7651	0.041*	
C149	0.2533 (3)	−0.2025 (3)	0.8380 (2)	0.0497 (15)	
H14J	0.2118	−0.2539	0.8285	0.075*	
H14K	0.2356	−0.1688	0.8632	0.075*	
H14L	0.3050	−0.2026	0.8545	0.075*	
C150	0.3289 (3)	−0.0959 (3)	0.8017 (2)	0.0529 (16)	
H15B	0.3347	−0.0798	0.7689	0.079*	
H15C	0.3811	−0.0955	0.8176	0.079*	
H15D	0.3122	−0.0612	0.8267	0.079*	

### Atomic displacement parameters (Å^2^)

**Table:**

	*U*^11^	*U*^22^	*U*^33^	*U*^12^	*U*^13^	*U*^23^
Sn1	0.01788 (14)	0.02831 (18)	0.01970 (14)	0.00772 (13)	0.00069 (11)	−0.00138 (12)
Sn2	0.01833 (15)	0.02719 (19)	0.02695 (16)	0.00804 (14)	−0.00101 (11)	−0.00219 (13)
Sn3	0.01755 (14)	0.02649 (18)	0.02081 (15)	0.00283 (13)	0.00295 (11)	0.00190 (12)
Sn4	0.01812 (15)	0.02110 (18)	0.03409 (18)	0.00446 (13)	0.00249 (12)	0.00342 (13)
Sn5	0.01811 (14)	0.02417 (18)	0.02256 (15)	0.00477 (13)	0.00327 (11)	0.00617 (12)
S1	0.0250 (5)	0.0235 (6)	0.0240 (5)	0.0097 (5)	−0.0020 (4)	−0.0021 (4)
S2	0.0230 (5)	0.0286 (7)	0.0257 (6)	0.0127 (5)	−0.0062 (4)	−0.0039 (5)
S3	0.0202 (5)	0.0249 (6)	0.0193 (5)	0.0076 (5)	−0.0023 (4)	0.0002 (4)
S4	0.0212 (5)	0.0254 (6)	0.0275 (6)	0.0095 (5)	−0.0002 (4)	0.0011 (5)
S5	0.0275 (6)	0.0233 (6)	0.0290 (6)	0.0119 (5)	−0.0027 (4)	−0.0033 (5)
S6	0.0206 (5)	0.0294 (7)	0.0259 (6)	0.0140 (5)	−0.0043 (4)	−0.0035 (5)
S7	0.0251 (5)	0.0255 (6)	0.0228 (5)	0.0085 (5)	−0.0039 (4)	0.0005 (4)
S8	0.0233 (5)	0.0273 (7)	0.0356 (6)	0.0097 (5)	−0.0034 (5)	−0.0008 (5)
S9	0.0309 (6)	0.0245 (6)	0.0254 (6)	0.0086 (5)	−0.0011 (4)	0.0033 (5)
S10	0.0216 (5)	0.0285 (7)	0.0238 (6)	0.0058 (5)	−0.0030 (4)	−0.0001 (5)
S11	0.0262 (6)	0.0259 (7)	0.0217 (5)	0.0058 (5)	−0.0041 (4)	0.0007 (4)
S12	0.0230 (6)	0.0327 (7)	0.0352 (6)	0.0102 (5)	−0.0018 (5)	−0.0030 (5)
S13	0.0259 (5)	0.0195 (6)	0.0229 (5)	0.0082 (5)	0.0000 (4)	−0.0005 (4)
S14	0.0276 (6)	0.0213 (6)	0.0281 (6)	0.0107 (5)	−0.0004 (4)	0.0021 (4)
S15	0.0311 (6)	0.0259 (7)	0.0291 (6)	0.0119 (5)	−0.0080 (5)	−0.0020 (5)
S16	0.0197 (6)	0.0284 (8)	0.0865 (11)	0.0064 (6)	−0.0082 (6)	0.0078 (7)
S17	0.0233 (5)	0.0214 (6)	0.0230 (5)	0.0090 (5)	−0.0005 (4)	0.0022 (4)
S18	0.0221 (5)	0.0235 (6)	0.0226 (5)	0.0091 (5)	0.0015 (4)	0.0036 (4)
S19	0.0322 (6)	0.0286 (7)	0.0225 (6)	0.0122 (5)	−0.0070 (4)	0.0012 (5)
S20	0.0213 (5)	0.0288 (7)	0.0306 (6)	0.0076 (5)	0.0000 (4)	0.0075 (5)
N1	0.0209 (18)	0.022 (2)	0.0219 (18)	0.0077 (16)	−0.0023 (14)	0.0068 (15)
N2	0.0160 (16)	0.025 (2)	0.0199 (18)	0.0057 (16)	0.0024 (13)	−0.0004 (14)
N3	0.0165 (16)	0.020 (2)	0.0255 (19)	0.0074 (15)	−0.0002 (14)	0.0057 (15)
N4	0.0151 (16)	0.022 (2)	0.0222 (18)	−0.0011 (15)	0.0003 (13)	−0.0033 (15)
N5	0.0228 (18)	0.026 (2)	0.029 (2)	0.0025 (17)	−0.0024 (15)	0.0083 (16)
N6	0.0178 (17)	0.029 (2)	0.0208 (18)	0.0013 (17)	0.0014 (14)	−0.0027 (15)
N7	0.0280 (19)	0.015 (2)	0.028 (2)	0.0054 (16)	−0.0032 (15)	0.0057 (15)
N8	0.026 (2)	0.028 (2)	0.025 (2)	−0.0029 (18)	−0.0026 (15)	0.0091 (16)
N9	0.0182 (17)	0.024 (2)	0.0233 (18)	0.0060 (16)	0.0009 (14)	0.0099 (15)
N10	0.0223 (18)	0.029 (2)	0.0223 (19)	0.0029 (17)	−0.0008 (15)	0.0051 (16)
C1	0.027 (2)	0.024 (3)	0.053 (3)	0.000 (2)	−0.010 (2)	0.012 (2)
C2	0.026 (3)	0.029 (3)	0.020 (3)	0.003 (2)	0.002 (3)	0.014 (3)
C3	0.037 (3)	0.032 (3)	0.034 (3)	0.012 (3)	0.009 (3)	0.007 (3)
C4	0.038 (3)	0.039 (3)	0.038 (5)	0.013 (3)	0.004 (4)	0.006 (3)
C2'	0.026 (3)	0.029 (3)	0.020 (3)	0.003 (2)	0.002 (3)	0.014 (3)
C3'	0.037 (3)	0.032 (3)	0.034 (3)	0.012 (3)	0.009 (3)	0.007 (3)
C4'	0.038 (3)	0.039 (3)	0.038 (5)	0.013 (3)	0.004 (4)	0.006 (3)
C5	0.022 (2)	0.039 (3)	0.025 (2)	0.011 (2)	0.0024 (18)	−0.003 (2)
C6	0.034 (3)	0.077 (4)	0.039 (3)	0.031 (3)	0.010 (2)	0.023 (3)
C7	0.033 (3)	0.046 (3)	0.042 (3)	0.014 (3)	0.014 (2)	0.006 (2)
C8	0.064 (4)	0.185 (9)	0.067 (5)	0.086 (5)	0.040 (4)	0.069 (5)
C9	0.022 (2)	0.013 (2)	0.0151 (19)	0.0038 (17)	0.0020 (15)	0.0041 (15)
C10	0.022 (2)	0.034 (3)	0.033 (2)	0.013 (2)	0.0012 (18)	0.012 (2)
C11	0.027 (2)	0.021 (3)	0.024 (2)	0.004 (2)	−0.0069 (18)	−0.0006 (18)
C12	0.034 (3)	0.026 (3)	0.052 (3)	0.010 (2)	−0.014 (2)	0.001 (2)
C13	0.050 (3)	0.028 (3)	0.064 (4)	0.010 (3)	−0.014 (3)	0.014 (3)
C14	0.064 (4)	0.028 (3)	0.040 (3)	0.001 (3)	−0.017 (3)	0.012 (2)
C15	0.052 (3)	0.034 (3)	0.031 (3)	0.004 (3)	0.008 (2)	0.009 (2)
C16	0.033 (3)	0.031 (3)	0.032 (3)	0.012 (2)	0.004 (2)	0.009 (2)
C17	0.024 (2)	0.029 (3)	0.026 (2)	0.005 (2)	−0.0072 (18)	0.0019 (19)
C18	0.027 (2)	0.043 (3)	0.039 (3)	0.005 (2)	−0.006 (2)	0.007 (2)
C19	0.040 (3)	0.046 (3)	0.027 (3)	0.008 (3)	0.007 (2)	−0.003 (2)
C20	0.0115 (18)	0.025 (2)	0.019 (2)	0.0062 (18)	0.0069 (15)	0.0081 (17)
C21	0.021 (2)	0.019 (2)	0.022 (2)	0.0034 (19)	0.0015 (16)	0.0021 (17)
C22	0.023 (2)	0.020 (2)	0.021 (2)	0.0091 (19)	0.0050 (16)	0.0077 (17)
C23	0.020 (2)	0.024 (3)	0.023 (2)	0.0070 (19)	0.0032 (16)	0.0057 (17)
C24	0.030 (2)	0.029 (3)	0.033 (3)	0.012 (2)	0.0137 (19)	0.016 (2)
C25	0.047 (3)	0.038 (3)	0.036 (3)	0.023 (3)	0.024 (2)	0.016 (2)
C26	0.058 (3)	0.027 (3)	0.032 (3)	0.018 (3)	0.019 (2)	0.003 (2)
C27	0.035 (3)	0.019 (3)	0.030 (2)	0.009 (2)	0.0084 (19)	0.0035 (19)
C28	0.019 (2)	0.035 (3)	0.023 (2)	0.010 (2)	−0.0044 (17)	0.0003 (19)
C29	0.059 (4)	0.109 (6)	0.025 (3)	0.056 (4)	0.000 (2)	0.020 (3)
C30	0.021 (3)	0.090 (5)	0.063 (4)	0.008 (3)	0.002 (2)	0.033 (4)
C31	0.024 (2)	0.036 (3)	0.033 (3)	0.009 (2)	0.0028 (19)	−0.003 (2)
C32	0.026 (2)	0.050 (3)	0.029 (3)	0.014 (2)	0.0018 (19)	−0.005 (2)
C33	0.041 (3)	0.049 (4)	0.048 (3)	0.017 (3)	0.019 (2)	0.003 (3)
C34	0.039 (3)	0.092 (5)	0.043 (3)	0.024 (3)	0.014 (3)	0.013 (3)
C35	0.026 (2)	0.028 (3)	0.047 (3)	0.005 (2)	−0.008 (2)	0.004 (2)
C36	0.033 (3)	0.051 (4)	0.074 (4)	0.014 (3)	0.000 (3)	0.022 (3)
C37	0.065 (4)	0.084 (6)	0.080 (5)	0.024 (4)	0.024 (4)	0.033 (4)
C38	0.056 (4)	0.035 (4)	0.094 (5)	0.022 (3)	0.022 (3)	0.014 (3)
C39	0.0139 (18)	0.020 (2)	0.019 (2)	0.0056 (17)	0.0037 (15)	0.0093 (16)
C40	0.020 (2)	0.032 (3)	0.035 (3)	0.015 (2)	0.0032 (18)	0.008 (2)
C41	0.023 (2)	0.024 (3)	0.023 (2)	0.009 (2)	−0.0032 (17)	0.0023 (18)
C42	0.037 (3)	0.038 (3)	0.036 (3)	0.020 (2)	0.003 (2)	0.009 (2)
C43	0.060 (4)	0.045 (4)	0.053 (4)	0.032 (3)	0.006 (3)	0.018 (3)
C44	0.060 (4)	0.038 (3)	0.042 (3)	0.017 (3)	−0.002 (3)	0.019 (2)
C45	0.042 (3)	0.044 (4)	0.042 (3)	0.010 (3)	0.009 (2)	0.020 (3)
C46	0.033 (3)	0.035 (3)	0.039 (3)	0.015 (2)	0.008 (2)	0.014 (2)
C47	0.024 (2)	0.026 (3)	0.022 (2)	0.009 (2)	−0.0016 (17)	0.0012 (18)
C48	0.039 (3)	0.044 (3)	0.029 (3)	0.010 (3)	0.009 (2)	0.004 (2)
C49	0.020 (2)	0.036 (3)	0.034 (3)	0.000 (2)	−0.0007 (18)	0.012 (2)
C50	0.0155 (19)	0.023 (2)	0.024 (2)	0.0050 (18)	0.0071 (16)	0.0115 (17)
C51	0.024 (2)	0.020 (2)	0.022 (2)	0.0004 (19)	0.0032 (17)	0.0061 (17)
C52	0.027 (2)	0.024 (3)	0.021 (2)	0.012 (2)	0.0029 (17)	0.0064 (17)
C53	0.027 (2)	0.030 (3)	0.028 (2)	0.006 (2)	0.0024 (18)	0.005 (2)
C54	0.030 (2)	0.038 (3)	0.037 (3)	0.009 (2)	0.009 (2)	0.018 (2)
C55	0.052 (3)	0.051 (4)	0.036 (3)	0.027 (3)	0.026 (2)	0.020 (2)
C56	0.057 (3)	0.036 (3)	0.036 (3)	0.011 (3)	0.020 (3)	0.000 (2)
C57	0.036 (3)	0.031 (3)	0.032 (3)	0.003 (2)	0.009 (2)	0.003 (2)
C58	0.019 (2)	0.048 (3)	0.028 (2)	0.009 (2)	−0.0017 (18)	0.001 (2)
C59	0.041 (3)	0.057 (4)	0.045 (3)	0.016 (3)	−0.004 (2)	0.026 (3)
C60	0.023 (3)	0.083 (5)	0.038 (3)	−0.009 (3)	−0.004 (2)	0.018 (3)
C61	0.020 (2)	0.034 (3)	0.026 (2)	0.008 (2)	0.0035 (17)	0.0063 (19)
C62	0.024 (2)	0.032 (3)	0.029 (2)	0.007 (2)	0.0051 (18)	0.003 (2)
C63	0.046 (3)	0.047 (4)	0.044 (3)	0.017 (3)	0.016 (2)	0.017 (3)
C64	0.053 (3)	0.034 (3)	0.065 (4)	0.018 (3)	0.021 (3)	0.017 (3)
C65	0.029 (2)	0.041 (3)	0.031 (3)	0.004 (2)	0.005 (2)	0.002 (2)
C66	0.048 (3)	0.058 (4)	0.052 (3)	0.030 (3)	0.020 (3)	0.025 (3)
C67	0.047 (3)	0.056 (4)	0.054 (4)	0.014 (3)	0.029 (3)	0.013 (3)
C68	0.071 (4)	0.154 (8)	0.071 (5)	0.076 (5)	0.043 (4)	0.054 (5)
C69	0.025 (2)	0.021 (2)	0.017 (2)	0.0063 (19)	0.0052 (16)	0.0118 (17)
C70	0.027 (2)	0.039 (3)	0.038 (3)	0.009 (2)	0.001 (2)	0.016 (2)
C71	0.027 (2)	0.029 (3)	0.028 (2)	0.004 (2)	−0.0077 (19)	0.005 (2)
C72	0.033 (3)	0.037 (3)	0.046 (3)	0.008 (2)	−0.011 (2)	0.006 (2)
C73	0.045 (3)	0.038 (4)	0.063 (4)	0.007 (3)	−0.021 (3)	0.016 (3)
C74	0.064 (4)	0.034 (4)	0.053 (4)	0.004 (3)	−0.020 (3)	0.018 (3)
C75	0.049 (3)	0.040 (4)	0.040 (3)	0.001 (3)	0.002 (2)	0.013 (3)
C76	0.038 (3)	0.032 (3)	0.037 (3)	0.009 (2)	−0.002 (2)	0.012 (2)
C77	0.026 (2)	0.026 (3)	0.032 (3)	−0.001 (2)	−0.0068 (19)	0.005 (2)
C78	0.058 (3)	0.042 (4)	0.030 (3)	0.006 (3)	−0.003 (2)	0.009 (2)
C79	0.046 (3)	0.039 (3)	0.038 (3)	0.002 (3)	0.002 (2)	0.008 (2)
C80	0.0060 (17)	0.027 (3)	0.022 (2)	−0.0017 (17)	0.0023 (15)	0.0072 (17)
C81	0.023 (2)	0.023 (3)	0.020 (2)	−0.001 (2)	0.0032 (17)	0.0041 (18)
C82	0.021 (2)	0.026 (3)	0.024 (2)	0.009 (2)	0.0036 (17)	0.0086 (18)
C83	0.034 (3)	0.033 (3)	0.041 (3)	0.010 (2)	0.014 (2)	0.005 (2)
C84	0.052 (3)	0.032 (3)	0.047 (3)	0.013 (3)	0.023 (3)	0.001 (2)
C85	0.050 (3)	0.046 (4)	0.053 (3)	0.024 (3)	0.032 (3)	0.015 (3)
C86	0.027 (2)	0.031 (3)	0.049 (3)	0.010 (2)	0.013 (2)	0.015 (2)
C87	0.023 (2)	0.024 (3)	0.033 (2)	0.010 (2)	0.0062 (18)	0.0089 (19)
C88	0.023 (2)	0.039 (3)	0.029 (2)	0.009 (2)	−0.0047 (19)	−0.004 (2)
C89	0.024 (3)	0.106 (6)	0.058 (4)	0.010 (3)	0.004 (3)	0.035 (4)
C90	0.055 (4)	0.139 (7)	0.031 (3)	0.059 (4)	0.004 (3)	0.030 (4)
C91	0.016 (2)	0.033 (3)	0.025 (2)	0.004 (2)	0.0045 (17)	0.0041 (19)
C92	0.024 (2)	0.024 (3)	0.024 (2)	0.001 (2)	0.0027 (17)	0.0066 (18)
C93	0.040 (3)	0.031 (3)	0.036 (3)	0.005 (2)	0.011 (2)	0.016 (2)
C94	0.059 (4)	0.040 (4)	0.056 (4)	0.014 (3)	0.014 (3)	0.025 (3)
C95	0.027 (3)	0.055 (4)	0.065 (4)	−0.011 (3)	0.019 (3)	−0.026 (3)
C96	0.079 (5)	0.051 (5)	0.078 (4)	0.003 (4)	0.061 (4)	0.022 (3)
C97	0.033 (3)	0.055 (4)	0.048 (4)	0.018 (3)	0.012 (3)	0.010 (3)
C98	0.066 (4)	0.074 (5)	0.073 (4)	0.032 (4)	0.024 (3)	0.021 (4)
C96'	0.079 (5)	0.051 (5)	0.078 (4)	0.003 (4)	0.061 (4)	0.022 (3)
C97'	0.033 (3)	0.055 (4)	0.048 (4)	0.018 (3)	0.012 (3)	0.010 (3)
C98'	0.066 (4)	0.074 (5)	0.073 (4)	0.032 (4)	0.024 (3)	0.021 (4)
C99	0.030 (2)	0.009 (2)	0.021 (2)	0.0050 (18)	−0.0039 (17)	0.0041 (16)
C100	0.023 (2)	0.027 (3)	0.032 (2)	0.012 (2)	0.0012 (18)	0.0086 (19)
C101	0.025 (2)	0.023 (2)	0.022 (2)	0.010 (2)	−0.0039 (17)	0.0034 (17)
C102	0.039 (3)	0.037 (3)	0.031 (3)	0.022 (2)	0.002 (2)	0.006 (2)
C103	0.061 (3)	0.035 (3)	0.041 (3)	0.029 (3)	0.002 (3)	0.012 (2)
C104	0.059 (3)	0.032 (3)	0.038 (3)	0.017 (3)	−0.001 (3)	0.017 (2)
C105	0.041 (3)	0.042 (3)	0.039 (3)	0.015 (3)	0.010 (2)	0.020 (2)
C106	0.037 (3)	0.036 (3)	0.034 (3)	0.019 (2)	0.011 (2)	0.015 (2)
C107	0.039 (3)	0.029 (3)	0.032 (3)	0.015 (2)	−0.014 (2)	−0.004 (2)
C108	0.067 (4)	0.056 (4)	0.039 (3)	−0.006 (3)	0.021 (3)	−0.014 (3)
C109	0.061 (4)	0.054 (4)	0.041 (3)	−0.014 (3)	0.012 (3)	0.005 (3)
C110	0.025 (2)	0.019 (3)	0.043 (3)	0.001 (2)	0.011 (2)	0.012 (2)
C111	0.043 (3)	0.025 (3)	0.025 (2)	0.002 (2)	−0.007 (2)	0.004 (2)
C112	0.046 (3)	0.036 (3)	0.030 (3)	0.014 (3)	−0.007 (2)	0.003 (2)
C113	0.067 (4)	0.044 (4)	0.034 (3)	0.013 (3)	−0.002 (3)	0.000 (3)
C114	0.081 (5)	0.058 (4)	0.036 (3)	0.023 (4)	0.004 (3)	−0.003 (3)
C115	0.069 (4)	0.076 (5)	0.043 (3)	0.045 (4)	0.017 (3)	0.017 (3)
C116	0.039 (3)	0.060 (4)	0.048 (3)	0.029 (3)	0.004 (2)	0.019 (3)
C117	0.038 (3)	0.045 (3)	0.034 (3)	0.020 (3)	−0.005 (2)	0.006 (2)
C118	0.022 (2)	0.064 (4)	0.034 (3)	0.004 (3)	0.000 (2)	0.023 (3)
C119	0.049 (3)	0.133 (7)	0.070 (4)	0.048 (4)	0.025 (3)	0.075 (5)
C120	0.029 (3)	0.082 (5)	0.046 (3)	−0.004 (3)	−0.005 (2)	−0.004 (3)
C121	0.024 (2)	0.056 (4)	0.027 (3)	0.000 (2)	0.0026 (19)	0.008 (2)
C122	0.029 (2)	0.038 (3)	0.034 (3)	0.012 (2)	0.012 (2)	0.011 (2)
C123	0.034 (3)	0.067 (4)	0.032 (3)	0.007 (3)	0.009 (2)	0.014 (3)
C124	0.042 (3)	0.099 (6)	0.038 (3)	0.023 (4)	0.017 (3)	0.018 (3)
C125	0.019 (2)	0.028 (3)	0.020 (2)	0.0017 (19)	0.0040 (16)	0.0096 (18)
C126	0.025 (2)	0.023 (3)	0.021 (2)	0.003 (2)	0.0063 (17)	0.0042 (17)
C127	0.038 (3)	0.034 (3)	0.032 (3)	0.012 (2)	0.010 (2)	0.013 (2)
C128	0.047 (3)	0.037 (3)	0.057 (4)	0.016 (3)	0.014 (3)	0.019 (3)
C129	0.0139 (18)	0.010 (2)	0.0171 (19)	0.0000 (16)	−0.0020 (14)	0.0095 (15)
C130	0.016 (2)	0.029 (3)	0.030 (2)	0.0083 (19)	−0.0004 (17)	0.0105 (19)
C131	0.021 (2)	0.026 (3)	0.022 (2)	0.006 (2)	−0.0021 (17)	0.0052 (18)
C132	0.027 (2)	0.033 (3)	0.034 (3)	0.010 (2)	0.0062 (19)	0.012 (2)
C133	0.037 (3)	0.043 (3)	0.042 (3)	0.008 (3)	0.004 (2)	0.018 (2)
C134	0.045 (3)	0.033 (3)	0.038 (3)	0.009 (3)	−0.006 (2)	0.013 (2)
C135	0.044 (3)	0.039 (3)	0.040 (3)	0.020 (3)	−0.006 (2)	0.004 (2)
C136	0.026 (2)	0.039 (3)	0.026 (2)	0.014 (2)	−0.0011 (18)	0.005 (2)
C137	0.028 (2)	0.027 (3)	0.022 (2)	0.002 (2)	−0.0033 (18)	0.0062 (19)
C138	0.044 (3)	0.044 (3)	0.027 (3)	0.007 (3)	−0.006 (2)	0.011 (2)
C139	0.044 (3)	0.035 (3)	0.036 (3)	−0.008 (3)	0.001 (2)	0.006 (2)
C140	0.020 (2)	0.023 (2)	0.023 (2)	0.0077 (19)	0.0065 (16)	0.0151 (17)
C141	0.038 (3)	0.021 (3)	0.030 (3)	0.003 (2)	−0.010 (2)	0.0036 (19)
C142	0.044 (3)	0.036 (3)	0.029 (3)	0.024 (3)	−0.003 (2)	0.004 (2)
C143	0.042 (3)	0.042 (3)	0.032 (3)	0.024 (3)	0.002 (2)	0.007 (2)
C144	0.056 (3)	0.074 (5)	0.046 (3)	0.042 (3)	0.019 (3)	0.021 (3)
C145	0.104 (6)	0.121 (7)	0.047 (4)	0.085 (6)	0.033 (4)	0.018 (4)
C146	0.099 (6)	0.089 (6)	0.047 (4)	0.050 (5)	0.010 (4)	−0.015 (4)
C147	0.066 (4)	0.058 (4)	0.039 (3)	0.030 (3)	−0.008 (3)	−0.014 (3)
C148	0.017 (2)	0.044 (3)	0.030 (3)	0.004 (2)	−0.0036 (18)	0.005 (2)
C149	0.040 (3)	0.073 (4)	0.042 (3)	0.024 (3)	−0.001 (2)	0.025 (3)
C150	0.031 (3)	0.057 (4)	0.044 (3)	−0.006 (3)	−0.004 (2)	0.000 (3)

### Geometric parameters (Å, °)

**Table:**

Sn1—C1	2.141 (5)	C59—H59B	0.9800
Sn1—C5	2.146 (4)	C59—H59C	0.9800
Sn1—S1	2.529 (1)	C60—H60A	0.9800
Sn1—S2	2.887 (1)	C60—H60B	0.9800
Sn1—S3	2.536 (1)	C60—H60C	0.9800
Sn1—S4	3.031 (1)	C61—C62	1.512 (4)
Sn2—C35	2.126 (5)	C61—H61A	0.9900
Sn2—C31	2.141 (4)	C61—H61B	0.9900
Sn2—S5	2.531 (1)	C62—C63	1.501 (4)
Sn2—S6	2.877 (1)	C62—H62A	0.9900
Sn2—S7	2.530 (1)	C62—H62B	0.9900
Sn2—S8	3.042 (1)	C63—C64	1.507 (4)
Sn3—C61	2.141 (4)	C63—H63A	0.9900
Sn3—C65	2.156 (4)	C63—H63B	0.9900
Sn3—S9	2.535 (1)	C64—H64A	0.9800
Sn3—S10	2.869 (1)	C64—H64B	0.9800
Sn3—S11	2.529 (1)	C64—H64C	0.9800
Sn3—S12	3.025 (1)	C65—C66	1.497 (4)
Sn4—C95	2.134 (4)	C65—H65A	0.9900
Sn4—C91	2.140 (4)	C65—H65B	0.9900
Sn4—S13	2.544 (1)	C66—C67	1.507 (4)
Sn4—S14	2.831 (1)	C66—H66A	0.9900
Sn4—S15	2.538 (1)	C66—H66B	0.9900
Sn4—S16	3.009 (1)	C67—C68	1.496 (4)
Sn5—C121	2.136 (4)	C67—H67A	0.9900
Sn5—C125	2.141 (4)	C67—H67B	0.9900
Sn5—S17	2.546 (1)	C68—H68A	0.9800
Sn5—S18	2.837 (1)	C68—H68B	0.9800
Sn5—S19	2.549 (1)	C68—H68C	0.9800
Sn5—S20	3.036 (1)	C70—C71	1.507 (7)
S1—C9	1.757 (4)	C70—H70A	0.9900
S2—C9	1.686 (4)	C70—H70B	0.9900
S3—C20	1.738 (4)	C71—C76	1.381 (7)
S4—C20	1.695 (4)	C71—C72	1.385 (6)
S5—C39	1.745 (4)	C72—C73	1.374 (7)
S6—C39	1.701 (4)	C72—H72	0.9500
S7—C50	1.736 (4)	C73—C74	1.370 (8)
S8—C50	1.688 (4)	C73—H73	0.9500
S9—C69	1.742 (4)	C74—C75	1.392 (8)
S10—C69	1.683 (4)	C74—H74	0.9500
S11—C80	1.760 (4)	C75—C76	1.388 (7)
S12—C80	1.682 (4)	C75—H75	0.9500
S13—C99	1.744 (4)	C76—H76	0.9500
S14—C99	1.699 (4)	C77—C78	1.506 (6)
S15—C110	1.731 (5)	C77—C79	1.528 (7)
S16—C110	1.706 (5)	C77—H77	1.0000
S17—C129	1.750 (4)	C78—H78A	0.9800
S18—C129	1.693 (4)	C78—H78B	0.9800
S19—C140	1.730 (4)	C78—H78C	0.9800
S20—C140	1.692 (4)	C79—H79A	0.9800
N1—C9	1.342 (5)	C79—H79B	0.9800
N1—C10	1.469 (5)	C79—H79C	0.9800
N1—C17	1.491 (5)	C81—C82	1.513 (6)
N2—C20	1.351 (5)	C81—H81A	0.9900
N2—C21	1.469 (5)	C81—H81B	0.9900
N2—C28	1.488 (5)	C82—C87	1.384 (6)
N3—C39	1.339 (4)	C82—C83	1.386 (6)
N3—C40	1.475 (5)	C83—C84	1.378 (6)
N3—C47	1.489 (5)	C83—H83	0.9500
N4—C50	1.356 (5)	C84—C85	1.381 (7)
N4—C51	1.469 (5)	C84—H84	0.9500
N4—C58	1.488 (5)	C85—C86	1.391 (7)
N5—C69	1.353 (5)	C85—H85	0.9500
N5—C70	1.474 (5)	C86—C87	1.379 (6)
N5—C77	1.493 (5)	C86—H86	0.9500
N6—C80	1.343 (5)	C87—H87	0.9500
N6—C81	1.459 (5)	C88—C89	1.488 (7)
N6—C88	1.496 (5)	C88—C90	1.490 (6)
N7—C99	1.345 (5)	C88—H88	1.0000
N7—C100	1.474 (5)	C89—H89A	0.9800
N7—C107	1.483 (5)	C89—H89B	0.9800
N8—C110	1.347 (5)	C89—H89C	0.9800
N8—C111	1.465 (6)	C90—H90A	0.9800
N8—C118	1.492 (5)	C90—H90B	0.9800
N9—C129	1.337 (4)	C90—H90C	0.9800
N9—C130	1.471 (5)	C91—C92	1.502 (4)
N9—C137	1.496 (5)	C91—H91A	0.9900
N10—C140	1.360 (5)	C91—H91B	0.9900
N10—C141	1.466 (5)	C92—C93	1.509 (4)
N10—C148	1.487 (5)	C92—H92A	0.9900
C1—C2'	1.495 (4)	C92—H92B	0.9900
C1—C2	1.539 (4)	C93—C94	1.510 (4)
C1—H1A	0.9900	C93—H93A	0.9900
C1—H1B	0.9900	C93—H93B	0.9900
C1—H1C	0.9900	C94—H94A	0.9800
C1—H1D	0.9900	C94—H94B	0.9800
C2—C3	1.505 (5)	C94—H94C	0.9800
C2—H2A	0.9900	C95—C96	1.476 (5)
C2—H2B	0.9900	C95—C96'	1.513 (5)
C3—C4	1.497 (5)	C95—H95A	0.9900
C3—H3A	0.9900	C95—H95B	0.9900
C3—H3B	0.9900	C95—H95C	0.9900
C4—H4A	0.9800	C95—H95D	0.9900
C4—H4B	0.9800	C96—C97	1.527 (5)
C4—H4C	0.9800	C96—H96A	0.9900
C2'—C3'	1.505 (5)	C96—H96B	0.9900
C2'—H2C	0.9900	C97—C98	1.494 (5)
C2'—H2D	0.9900	C97—H97A	0.9900
C3'—C4'	1.499 (5)	C97—H97B	0.9900
C3'—H3C	0.9900	C98—H98A	0.9800
C3'—H3D	0.9900	C98—H98B	0.9800
C4'—H4D	0.9800	C98—H98C	0.9800
C4'—H4E	0.9800	C96'—C97'	1.504 (5)
C4'—H4F	0.9800	C96'—H96C	0.9900
C5—C6	1.501 (4)	C96'—H96D	0.9900
C5—H5A	0.9900	C97'—C98'	1.502 (5)
C5—H5B	0.9900	C97'—H97C	0.9900
C6—C7	1.500 (4)	C97'—H97D	0.9900
C6—H6A	0.9900	C98'—H98D	0.9800
C6—H6B	0.9900	C98'—H98E	0.9800
C7—C8	1.494 (4)	C98'—H98F	0.9800
C7—H7A	0.9900	C100—C101	1.507 (6)
C7—H7B	0.9900	C100—H10C	0.9900
C8—H8A	0.9800	C100—H10D	0.9900
C8—H8B	0.9800	C101—C102	1.388 (6)
C8—H8C	0.9800	C101—C106	1.388 (6)
C10—C11	1.516 (6)	C102—C103	1.374 (7)
C10—H10A	0.9900	C102—H10E	0.9500
C10—H10B	0.9900	C103—C104	1.384 (7)
C11—C16	1.385 (6)	C103—H10F	0.9500
C11—C12	1.399 (6)	C104—C105	1.377 (7)
C12—C13	1.379 (7)	C104—H10G	0.9500
C12—H12	0.9500	C105—C106	1.372 (7)
C13—C14	1.378 (8)	C105—H10H	0.9500
C13—H13	0.9500	C106—H10I	0.9500
C14—C15	1.374 (7)	C107—C108	1.475 (7)
C14—H14	0.9500	C107—C109	1.534 (7)
C15—C16	1.395 (7)	C107—H10J	1.0000
C15—H15	0.9500	C108—H10K	0.9800
C16—H16	0.9500	C108—H10L	0.9800
C17—C19	1.507 (6)	C108—H10M	0.9800
C17—C18	1.531 (6)	C109—H10N	0.9800
C17—H17	1.0000	C109—H10O	0.9800
C18—H18A	0.9800	C109—H10P	0.9800
C18—H18B	0.9800	C111—C112	1.511 (7)
C18—H18C	0.9800	C111—H11A	0.9900
C19—H19A	0.9800	C111—H11B	0.9900
C19—H19B	0.9800	C112—C113	1.383 (6)
C19—H19C	0.9800	C112—C117	1.385 (7)
C21—C22	1.522 (5)	C113—C114	1.373 (8)
C21—H21A	0.9900	C113—H11C	0.9500
C21—H21B	0.9900	C114—C115	1.359 (8)
C22—C27	1.387 (5)	C114—H11D	0.9500
C22—C23	1.387 (6)	C115—C116	1.396 (7)
C23—C24	1.386 (6)	C115—H11E	0.9500
C23—H23	0.9500	C116—C117	1.373 (7)
C24—C25	1.378 (6)	C116—H11F	0.9500
C24—H24	0.9500	C117—H11G	0.9500
C25—C26	1.381 (7)	C118—C119	1.517 (7)
C25—H25	0.9500	C118—C120	1.523 (7)
C26—C27	1.378 (6)	C118—H11H	1.0000
C26—H26	0.9500	C119—H11I	0.9800
C27—H27	0.9500	C119—H11J	0.9800
C28—C30	1.494 (7)	C119—H11K	0.9800
C28—C29	1.499 (6)	C120—H12B	0.9800
C28—H28	1.0000	C120—H12C	0.9800
C29—H29A	0.9800	C120—H12D	0.9800
C29—H29B	0.9800	C121—C122	1.503 (4)
C29—H29C	0.9800	C121—H12E	0.9900
C30—H30A	0.9800	C121—H12V	0.9900
C30—H30B	0.9800	C122—C123	1.498 (4)
C30—H30C	0.9800	C122—H12F	0.9900
C31—C32	1.514 (4)	C122—H12G	0.9900
C31—H31A	0.9900	C123—C124	1.494 (4)
C31—H31B	0.9900	C123—H12H	0.9900
C32—C33	1.515 (4)	C123—H12I	0.9900
C32—H32A	0.9900	C124—H12J	0.9800
C32—H32B	0.9900	C124—H12K	0.9800
C33—C34	1.503 (4)	C124—H12L	0.9800
C33—H33A	0.9900	C125—C126	1.506 (4)
C33—H33B	0.9900	C125—H12M	0.9900
C34—H34A	0.9800	C125—H12N	0.9900
C34—H34B	0.9800	C126—C127	1.511 (4)
C34—H34C	0.9800	C126—H12O	0.9900
C35—C36	1.522 (4)	C126—H12P	0.9900
C35—H35A	0.9900	C127—C128	1.508 (4)
C35—H35B	0.9900	C127—H12Q	0.9900
C36—C37	1.481 (4)	C127—H12R	0.9900
C36—H36A	0.9900	C128—H12S	0.9800
C36—H36B	0.9900	C128—H12T	0.9800
C37—C38	1.510 (4)	C128—H12U	0.9800
C37—H37A	0.9900	C130—C131	1.516 (6)
C37—H37B	0.9900	C130—H13B	0.9900
C38—H38A	0.9800	C130—H13C	0.9900
C38—H38B	0.9800	C131—C132	1.383 (6)
C38—H38C	0.9800	C131—C136	1.389 (6)
C40—C41	1.504 (6)	C132—C133	1.382 (7)
C40—H40A	0.9900	C132—H13D	0.9500
C40—H40B	0.9900	C133—C134	1.388 (7)
C41—C46	1.382 (6)	C133—H13E	0.9500
C41—C42	1.397 (6)	C134—C135	1.375 (7)
C42—C43	1.382 (7)	C134—H13F	0.9500
C42—H42	0.9500	C135—C136	1.382 (7)
C43—C44	1.368 (7)	C135—H13G	0.9500
C43—H43	0.9500	C136—H13H	0.9500
C44—C45	1.374 (7)	C137—C139	1.508 (6)
C44—H44	0.9500	C137—C138	1.512 (6)
C45—C46	1.398 (7)	C137—H13I	1.0000
C45—H45	0.9500	C138—H13J	0.9800
C46—H46	0.9500	C138—H13K	0.9800
C47—C48	1.516 (6)	C138—H13L	0.9800
C47—C49	1.532 (5)	C139—H13M	0.9800
C47—H47	1.0000	C139—H13N	0.9800
C48—H48A	0.9800	C139—H13O	0.9800
C48—H48B	0.9800	C141—C142	1.502 (7)
C48—H48C	0.9800	C141—H14B	0.9900
C49—H49A	0.9800	C141—H14C	0.9900
C49—H49B	0.9800	C142—C143	1.386 (7)
C49—H49C	0.9800	C142—C147	1.391 (6)
C51—C52	1.514 (6)	C143—C144	1.381 (7)
C51—H51A	0.9900	C143—H14D	0.9500
C51—H51B	0.9900	C144—C145	1.385 (8)
C52—C57	1.390 (6)	C144—H14E	0.9500
C52—C53	1.392 (6)	C145—C146	1.376 (10)
C53—C54	1.373 (6)	C145—H14F	0.9500
C53—H53	0.9500	C146—C147	1.379 (9)
C54—C55	1.389 (7)	C146—H14G	0.9500
C54—H54	0.9500	C147—H14H	0.9500
C55—C56	1.374 (7)	C148—C149	1.495 (7)
C55—H55	0.9500	C148—C150	1.517 (7)
C56—C57	1.388 (6)	C148—H14I	1.0000
C56—H56	0.9500	C149—H14J	0.9800
C57—H57	0.9500	C149—H14K	0.9800
C58—C59	1.485 (7)	C149—H14L	0.9800
C58—C60	1.509 (7)	C150—H15B	0.9800
C58—H58	1.0000	C150—H15C	0.9800
C59—H59A	0.9800	C150—H15D	0.9800
			
C1—Sn1—C5	129.0 (2)	C63—C62—C61	113.9 (3)
C1—Sn1—S1	108.14 (11)	C63—C62—H62A	108.8
C5—Sn1—S1	115.24 (11)	C61—C62—H62A	108.8
C1—Sn1—S3	105.81 (13)	C63—C62—H62B	108.8
C5—Sn1—S3	106.94 (13)	C61—C62—H62B	108.8
S1—Sn1—S3	79.95 (3)	H62A—C62—H62B	107.7
C1—Sn1—S2	86.46 (13)	C62—C63—C64	113.9 (4)
C5—Sn1—S2	87.93 (11)	C62—C63—H63A	108.8
S1—Sn1—S2	65.65 (3)	C64—C63—H63A	108.8
S3—Sn1—S2	145.60 (3)	C62—C63—H63B	108.8
C1—Sn1—S4	80.36 (12)	C64—C63—H63B	108.8
C5—Sn1—S4	80.46 (11)	H63A—C63—H63B	107.7
S1—Sn1—S4	143.47 (3)	C63—C64—H64A	109.5
S3—Sn1—S4	63.67 (3)	C63—C64—H64B	109.5
S2—Sn1—S4	150.70 (3)	H64A—C64—H64B	109.5
C31—Sn2—C35	128.4 (2)	C63—C64—H64C	109.5
C35—Sn2—S7	106.00 (12)	H64A—C64—H64C	109.5
C31—Sn2—S7	107.25 (13)	H64B—C64—H64C	109.5
C35—Sn2—S5	108.31 (11)	C66—C65—Sn3	119.0 (3)
C31—Sn2—S5	114.79 (12)	C66—C65—H65A	107.6
S7—Sn2—S5	81.23 (3)	Sn3—C65—H65A	107.6
C35—Sn2—S6	85.55 (13)	C66—C65—H65B	107.6
C31—Sn2—S6	87.23 (12)	Sn3—C65—H65B	107.6
S7—Sn2—S6	147.15 (3)	H65A—C65—H65B	107.0
S5—Sn2—S6	65.92 (3)	C65—C66—C67	112.3 (4)
C35—Sn2—S8	79.77 (12)	C65—C66—H66A	109.1
C31—Sn2—S8	81.11 (11)	C67—C66—H66A	109.1
S7—Sn2—S8	63.56 (3)	C65—C66—H66B	109.1
S5—Sn2—S8	144.60 (3)	C67—C66—H66B	109.1
S6—Sn2—S8	149.20 (3)	H66A—C66—H66B	107.9
C61—Sn3—C65	127.1 (2)	C68—C67—C66	114.6 (4)
C61—Sn3—S11	107.63 (11)	C68—C67—H67A	108.6
C65—Sn3—S11	106.19 (14)	C66—C67—H67A	108.6
C61—Sn3—S9	108.84 (10)	C68—C67—H67B	108.6
C65—Sn3—S9	115.46 (12)	C66—C67—H67B	108.6
S11—Sn3—S9	81.45 (3)	H67A—C67—H67B	107.6
C61—Sn3—S10	84.20 (12)	C67—C68—H68A	109.5
C65—Sn3—S10	88.60 (12)	C67—C68—H68B	109.5
S11—Sn3—S10	147.29 (3)	H68A—C68—H68B	109.5
S9—Sn3—S10	65.84 (3)	C67—C68—H68C	109.5
C61—Sn3—S12	80.57 (11)	H68A—C68—H68C	109.5
C65—Sn3—S12	79.22 (11)	H68B—C68—H68C	109.5
S11—Sn3—S12	63.97 (3)	N5—C69—S10	122.2 (3)
S9—Sn3—S12	145.29 (3)	N5—C69—S9	118.8 (3)
S10—Sn3—S12	148.67 (3)	S10—C69—S9	119.0 (2)
C91—Sn4—C95	136.9 (2)	N5—C70—C71	116.0 (4)
C95—Sn4—S15	103.13 (15)	N5—C70—H70A	108.3
C91—Sn4—S15	102.40 (11)	C71—C70—H70A	108.3
C95—Sn4—S13	110.33 (16)	N5—C70—H70B	108.3
C91—Sn4—S13	106.98 (10)	C71—C70—H70B	108.3
S15—Sn4—S13	82.49 (3)	H70A—C70—H70B	107.4
C95—Sn4—S14	90.28 (14)	C76—C71—C72	119.3 (5)
C91—Sn4—S14	85.44 (11)	C76—C71—C70	122.9 (4)
S15—Sn4—S14	148.73 (3)	C72—C71—C70	117.8 (4)
S13—Sn4—S14	66.32 (3)	C73—C72—C71	119.9 (5)
C95—Sn4—S16	81.52 (15)	C73—C72—H72	120.0
C91—Sn4—S16	79.25 (11)	C71—C72—H72	120.0
S15—Sn4—S16	64.01 (4)	C74—C73—C72	121.1 (5)
S13—Sn4—S16	146.39 (4)	C74—C73—H73	119.5
S14—Sn4—S16	146.91 (3)	C72—C73—H73	119.5
C121—Sn5—C125	135.6 (2)	C73—C74—C75	119.7 (6)
C121—Sn5—S17	110.68 (13)	C73—C74—H74	120.1
C125—Sn5—S17	108.94 (10)	C75—C74—H74	120.1
C121—Sn5—S19	101.62 (14)	C76—C75—C74	119.1 (5)
C125—Sn5—S19	102.85 (11)	C76—C75—H75	120.4
S17—Sn5—S19	82.03 (3)	C74—C75—H75	120.4
C121—Sn5—S18	92.04 (12)	C71—C76—C75	120.8 (5)
C125—Sn5—S18	86.15 (11)	C71—C76—H76	119.6
S17—Sn5—S18	66.22 (3)	C75—C76—H76	119.6
S19—Sn5—S18	148.17 (3)	N5—C77—C78	113.2 (4)
C121—Sn5—S20	79.36 (11)	N5—C77—C79	109.9 (4)
C125—Sn5—S20	79.39 (11)	C78—C77—C79	111.7 (4)
S17—Sn5—S20	145.39 (3)	N5—C77—H77	107.2
S19—Sn5—S20	63.38 (3)	C78—C77—H77	107.2
S18—Sn5—S20	148.23 (3)	C79—C77—H77	107.2
C9—S1—Sn1	93.06 (13)	C77—C78—H78A	109.5
C9—S2—Sn1	82.79 (13)	C77—C78—H78B	109.5
C20—S3—Sn1	96.11 (14)	H78A—C78—H78B	109.5
C20—S4—Sn1	80.66 (13)	C77—C78—H78C	109.5
C39—S5—Sn2	92.80 (12)	H78A—C78—H78C	109.5
C39—S6—Sn2	82.45 (13)	H78B—C78—H78C	109.5
C50—S7—Sn2	95.88 (14)	C77—C79—H79A	109.5
C50—S8—Sn2	79.96 (14)	C77—C79—H79B	109.5
C69—S9—Sn3	92.46 (13)	H79A—C79—H79B	109.5
C69—S10—Sn3	82.71 (14)	C77—C79—H79C	109.5
C80—S11—Sn3	95.66 (14)	H79A—C79—H79C	109.5
C80—S12—Sn3	80.91 (13)	H79B—C79—H79C	109.5
C99—S13—Sn4	92.06 (13)	N6—C80—S12	123.5 (3)
C99—S14—Sn4	83.62 (13)	N6—C80—S11	117.1 (3)
C110—S15—Sn4	95.72 (16)	S12—C80—S11	119.4 (2)
C110—S16—Sn4	80.73 (16)	N6—C81—C82	114.1 (4)
C129—S17—Sn5	92.08 (12)	N6—C81—H81A	108.7
C129—S18—Sn5	83.75 (13)	C82—C81—H81A	108.7
C140—S19—Sn5	95.61 (14)	N6—C81—H81B	108.7
C140—S20—Sn5	80.31 (14)	C82—C81—H81B	108.7
C9—N1—C10	120.9 (3)	H81A—C81—H81B	107.6
C9—N1—C17	119.6 (3)	C87—C82—C83	119.0 (4)
C10—N1—C17	119.5 (3)	C87—C82—C81	121.5 (4)
C20—N2—C21	122.0 (3)	C83—C82—C81	119.6 (4)
C20—N2—C28	121.3 (3)	C84—C83—C82	120.7 (5)
C21—N2—C28	116.5 (3)	C84—C83—H83	119.7
C39—N3—C40	120.7 (3)	C82—C83—H83	119.7
C39—N3—C47	120.4 (3)	C83—C84—C85	120.0 (5)
C40—N3—C47	118.9 (3)	C83—C84—H84	120.0
C50—N4—C51	121.5 (3)	C85—C84—H84	120.0
C50—N4—C58	120.4 (3)	C84—C85—C86	119.9 (5)
C51—N4—C58	118.1 (3)	C84—C85—H85	120.1
C69—N5—C70	121.2 (4)	C86—C85—H85	120.1
C69—N5—C77	120.2 (4)	C87—C86—C85	119.5 (5)
C70—N5—C77	118.4 (3)	C87—C86—H86	120.2
C80—N6—C81	122.8 (3)	C85—C86—H86	120.2
C80—N6—C88	120.0 (4)	C86—C87—C82	120.9 (4)
C81—N6—C88	117.1 (3)	C86—C87—H87	119.5
C99—N7—C100	120.3 (3)	C82—C87—H87	119.5
C99—N7—C107	120.8 (3)	C89—C88—C90	112.4 (5)
C100—N7—C107	118.9 (3)	C89—C88—N6	110.5 (4)
C110—N8—C111	120.9 (4)	C90—C88—N6	112.8 (4)
C110—N8—C118	121.0 (4)	C89—C88—H88	106.9
C111—N8—C118	118.1 (4)	C90—C88—H88	106.9
C129—N9—C130	121.3 (3)	N6—C88—H88	106.9
C129—N9—C137	120.2 (3)	C88—C89—H89A	109.5
C130—N9—C137	118.2 (3)	C88—C89—H89B	109.5
C140—N10—C141	121.4 (3)	H89A—C89—H89B	109.5
C140—N10—C148	120.9 (4)	C88—C89—H89C	109.5
C141—N10—C148	117.7 (4)	H89A—C89—H89C	109.5
C2'—C1—Sn1	128.1 (5)	H89B—C89—H89C	109.5
C2—C1—Sn1	102.3 (3)	C88—C90—H90A	109.5
C2—C1—H1A	111.3	C88—C90—H90B	109.5
Sn1—C1—H1A	111.3	H90A—C90—H90B	109.5
C2'—C1—H1B	108.4	C88—C90—H90C	109.5
C2—C1—H1B	111.3	H90A—C90—H90C	109.5
Sn1—C1—H1B	111.3	H90B—C90—H90C	109.5
H1A—C1—H1B	109.2	C92—C91—Sn4	114.7 (3)
C2'—C1—H1C	105.3	C92—C91—H91A	108.6
C2—C1—H1C	133.9	Sn4—C91—H91A	108.6
Sn1—C1—H1C	105.3	C92—C91—H91B	108.6
C2'—C1—H1D	105.3	Sn4—C91—H91B	108.6
C2—C1—H1D	101.5	H91A—C91—H91B	107.6
Sn1—C1—H1D	105.3	C91—C92—C93	113.3 (3)
H1C—C1—H1D	106.0	C91—C92—H92A	108.9
C3—C2—C1	111.9 (5)	C93—C92—H92A	108.9
C3—C2—H2A	109.2	C91—C92—H92B	108.9
C1—C2—H2A	109.2	C93—C92—H92B	108.9
C3—C2—H2B	109.2	H92A—C92—H92B	107.7
C1—C2—H2B	109.2	C94—C93—C92	113.8 (4)
H2A—C2—H2B	107.9	C94—C93—H93A	108.8
C4—C3—C2	115.8 (6)	C92—C93—H93A	108.8
C4—C3—H3A	108.3	C94—C93—H93B	108.8
C2—C3—H3A	108.3	C92—C93—H93B	108.8
C4—C3—H3B	108.3	H93A—C93—H93B	107.7
C2—C3—H3B	108.3	C93—C94—H94A	109.5
H3A—C3—H3B	107.4	C93—C94—H94B	109.5
C3—C4—H4A	109.5	H94A—C94—H94B	109.5
C3—C4—H4B	109.5	C93—C94—H94C	109.5
H4A—C4—H4B	109.5	H94A—C94—H94C	109.5
C3—C4—H4C	109.5	H94B—C94—H94C	109.5
H4A—C4—H4C	109.5	C96—C95—Sn4	126.3 (4)
H4B—C4—H4C	109.5	C96'—C95—Sn4	113.6 (4)
C1—C2'—C3'	109.4 (5)	C96—C95—H95A	105.7
C1—C2'—H2C	109.8	C96'—C95—H95A	95.5
C3'—C2'—H2C	109.8	Sn4—C95—H95A	105.7
C1—C2'—H2D	109.8	C96—C95—H95B	105.7
C3'—C2'—H2D	109.8	Sn4—C95—H95B	105.7
H2C—C2'—H2D	108.3	H95A—C95—H95B	106.2
C4'—C3'—C2'	114.5 (6)	C96—C95—H95C	114.1
C4'—C3'—H3C	108.6	C96'—C95—H95C	108.9
C2'—C3'—H3C	108.6	Sn4—C95—H95C	108.9
C4'—C3'—H3D	108.6	C96'—C95—H95D	108.9
C2'—C3'—H3D	108.6	Sn4—C95—H95D	108.9
H3C—C3'—H3D	107.6	H95C—C95—H95D	107.7
C3'—C4'—H4D	109.5	C95—C96—C97	107.2 (5)
C3'—C4'—H4E	109.5	C95—C96—H96A	110.3
H4D—C4'—H4E	109.5	C97—C96—H96A	110.3
C3'—C4'—H4F	109.5	C95—C96—H96B	110.3
H4D—C4'—H4F	109.5	C97—C96—H96B	110.3
H4E—C4'—H4F	109.5	H96A—C96—H96B	108.5
C6—C5—Sn1	119.0 (3)	C98—C97—C96	110.9 (6)
C6—C5—H5A	107.6	C98—C97—H97A	109.5
Sn1—C5—H5A	107.6	C96—C97—H97A	109.5
C6—C5—H5B	107.6	C98—C97—H97B	109.5
Sn1—C5—H5B	107.6	C96—C97—H97B	109.5
H5A—C5—H5B	107.0	H97A—C97—H97B	108.0
C7—C6—C5	112.9 (3)	C97—C98—H98A	109.5
C7—C6—H6A	109.0	C97—C98—H98B	109.5
C5—C6—H6A	109.0	H98A—C98—H98B	109.5
C7—C6—H6B	109.0	C97—C98—H98C	109.5
C5—C6—H6B	109.0	H98A—C98—H98C	109.5
H6A—C6—H6B	107.8	H98B—C98—H98C	109.5
C8—C7—C6	117.4 (4)	C97'—C96'—C95	120.4 (7)
C8—C7—H7A	108.0	C97'—C96'—H96C	107.2
C6—C7—H7A	108.0	C95—C96'—H96C	107.2
C8—C7—H7B	108.0	C97'—C96'—H96D	107.2
C6—C7—H7B	108.0	C95—C96'—H96D	107.2
H7A—C7—H7B	107.2	H96C—C96'—H96D	106.9
C7—C8—H8A	109.5	C98'—C97'—C96'	118.2 (10)
C7—C8—H8B	109.5	C98'—C97'—H97C	107.7
H8A—C8—H8B	109.5	C96'—C97'—H97C	107.7
C7—C8—H8C	109.5	C98'—C97'—H97D	107.7
H8A—C8—H8C	109.5	C96'—C97'—H97D	107.7
H8B—C8—H8C	109.5	H97C—C97'—H97D	107.1
N1—C9—S2	123.9 (3)	C97'—C98'—H98D	109.5
N1—C9—S1	118.0 (3)	C97'—C98'—H98E	109.5
S2—C9—S1	118.0 (2)	H98D—C98'—H98E	109.5
N1—C10—C11	115.2 (4)	C97'—C98'—H98F	109.5
N1—C10—H10A	108.5	H98D—C98'—H98F	109.5
C11—C10—H10A	108.5	H98E—C98'—H98F	109.5
N1—C10—H10B	108.5	N7—C99—S14	122.8 (3)
C11—C10—H10B	108.5	N7—C99—S13	119.1 (3)
H10A—C10—H10B	107.5	S14—C99—S13	117.9 (2)
C16—C11—C12	118.6 (5)	N7—C100—C101	115.1 (4)
C16—C11—C10	124.1 (4)	N7—C100—H10C	108.5
C12—C11—C10	117.3 (4)	C101—C100—H10C	108.5
C13—C12—C11	120.3 (5)	N7—C100—H10D	108.5
C13—C12—H12	119.9	C101—C100—H10D	108.5
C11—C12—H12	119.9	H10C—C100—H10D	107.5
C14—C13—C12	120.7 (5)	C102—C101—C106	118.2 (4)
C14—C13—H13	119.7	C102—C101—C100	117.1 (4)
C12—C13—H13	119.7	C106—C101—C100	124.6 (4)
C15—C14—C13	119.9 (5)	C103—C102—C101	120.4 (5)
C15—C14—H14	120.1	C103—C102—H10E	119.8
C13—C14—H14	120.1	C101—C102—H10E	119.8
C14—C15—C16	120.0 (5)	C102—C103—C104	120.8 (5)
C14—C15—H15	120.0	C102—C103—H10F	119.6
C16—C15—H15	120.0	C104—C103—H10F	119.6
C11—C16—C15	120.6 (4)	C105—C104—C103	119.1 (5)
C11—C16—H16	119.7	C105—C104—H10G	120.5
C15—C16—H16	119.7	C103—C104—H10G	120.5
N1—C17—C19	111.4 (4)	C106—C105—C104	120.2 (5)
N1—C17—C18	112.5 (4)	C106—C105—H10H	119.9
C19—C17—C18	110.7 (4)	C104—C105—H10H	119.9
N1—C17—H17	107.3	C105—C106—C101	121.2 (4)
C19—C17—H17	107.3	C105—C106—H10I	119.4
C18—C17—H17	107.3	C101—C106—H10I	119.4
C17—C18—H18A	109.5	C108—C107—N7	112.5 (4)
C17—C18—H18B	109.5	C108—C107—C109	109.7 (4)
H18A—C18—H18B	109.5	N7—C107—C109	112.2 (4)
C17—C18—H18C	109.5	C108—C107—H10J	107.4
H18A—C18—H18C	109.5	N7—C107—H10J	107.4
H18B—C18—H18C	109.5	C109—C107—H10J	107.4
C17—C19—H19A	109.5	C107—C108—H10K	109.5
C17—C19—H19B	109.5	C107—C108—H10L	109.5
H19A—C19—H19B	109.5	H10K—C108—H10L	109.5
C17—C19—H19C	109.5	C107—C108—H10M	109.5
H19A—C19—H19C	109.5	H10K—C108—H10M	109.5
H19B—C19—H19C	109.5	H10L—C108—H10M	109.5
N2—C20—S4	122.2 (3)	C107—C109—H10N	109.5
N2—C20—S3	118.2 (3)	C107—C109—H10O	109.5
S4—C20—S3	119.6 (2)	H10N—C109—H10O	109.5
N2—C21—C22	113.8 (4)	C107—C109—H10P	109.5
N2—C21—H21A	108.8	H10N—C109—H10P	109.5
C22—C21—H21A	108.8	H10O—C109—H10P	109.5
N2—C21—H21B	108.8	N8—C110—S16	120.9 (3)
C22—C21—H21B	108.8	N8—C110—S15	119.6 (3)
H21A—C21—H21B	107.7	S16—C110—S15	119.4 (3)
C27—C22—C23	118.9 (4)	N8—C111—C112	114.5 (4)
C27—C22—C21	119.6 (4)	N8—C111—H11A	108.6
C23—C22—C21	121.5 (4)	C112—C111—H11A	108.6
C24—C23—C22	120.6 (4)	N8—C111—H11B	108.6
C24—C23—H23	119.7	C112—C111—H11B	108.6
C22—C23—H23	119.7	H11A—C111—H11B	107.6
C25—C24—C23	119.8 (4)	C113—C112—C117	118.6 (5)
C25—C24—H24	120.1	C113—C112—C111	119.9 (5)
C23—C24—H24	120.1	C117—C112—C111	121.5 (4)
C24—C25—C26	119.9 (4)	C114—C113—C112	120.7 (6)
C24—C25—H25	120.1	C114—C113—H11C	119.7
C26—C25—H25	120.1	C112—C113—H11C	119.7
C27—C26—C25	120.3 (4)	C115—C114—C113	120.7 (5)
C27—C26—H26	119.8	C115—C114—H11D	119.6
C25—C26—H26	119.8	C113—C114—H11D	119.6
C26—C27—C22	120.4 (4)	C114—C115—C116	119.5 (6)
C26—C27—H27	119.8	C114—C115—H11E	120.3
C22—C27—H27	119.8	C116—C115—H11E	120.3
N2—C28—C30	110.5 (4)	C117—C116—C115	119.8 (6)
N2—C28—C29	112.2 (3)	C117—C116—H11F	120.1
C30—C28—C29	112.9 (4)	C115—C116—H11F	120.1
N2—C28—H28	107.0	C116—C117—C112	120.7 (5)
C30—C28—H28	107.0	C116—C117—H11G	119.7
C29—C28—H28	107.0	C112—C117—H11G	119.7
C28—C29—H29A	109.5	N8—C118—C119	110.9 (4)
C28—C29—H29B	109.5	N8—C118—C120	110.6 (4)
H29A—C29—H29B	109.5	C119—C118—C120	111.5 (5)
C28—C29—H29C	109.5	N8—C118—H11H	107.9
H29A—C29—H29C	109.5	C119—C118—H11H	107.9
H29B—C29—H29C	109.5	C120—C118—H11H	107.9
C28—C30—H30A	109.5	C118—C119—H11I	109.5
C28—C30—H30B	109.5	C118—C119—H11J	109.5
H30A—C30—H30B	109.5	H11I—C119—H11J	109.5
C28—C30—H30C	109.5	C118—C119—H11K	109.5
H30A—C30—H30C	109.5	H11I—C119—H11K	109.5
H30B—C30—H30C	109.5	H11J—C119—H11K	109.5
C32—C31—Sn2	118.2 (3)	C118—C120—H12B	109.5
C32—C31—H31A	107.8	C118—C120—H12C	109.5
Sn2—C31—H31A	107.8	H12B—C120—H12C	109.5
C32—C31—H31B	107.8	C118—C120—H12D	109.5
Sn2—C31—H31B	107.8	H12B—C120—H12D	109.5
H31A—C31—H31B	107.1	H12C—C120—H12D	109.5
C31—C32—C33	110.7 (3)	C122—C121—Sn5	122.5 (3)
C31—C32—H32A	109.5	C122—C121—H12E	106.7
C33—C32—H32A	109.5	Sn5—C121—H12E	106.7
C31—C32—H32B	109.5	C122—C121—H12V	106.7
C33—C32—H32B	109.5	Sn5—C121—H12V	106.7
H32A—C32—H32B	108.1	H12E—C121—H12V	106.6
C34—C33—C32	114.7 (4)	C123—C122—C121	112.3 (3)
C34—C33—H33A	108.6	C123—C122—H12F	109.1
C32—C33—H33A	108.6	C121—C122—H12F	109.1
C34—C33—H33B	108.6	C123—C122—H12G	109.1
C32—C33—H33B	108.6	C121—C122—H12G	109.1
H33A—C33—H33B	107.6	H12F—C122—H12G	107.9
C33—C34—H34A	109.5	C124—C123—C122	116.5 (4)
C33—C34—H34B	109.5	C124—C123—H12H	108.2
H34A—C34—H34B	109.5	C122—C123—H12H	108.2
C33—C34—H34C	109.5	C124—C123—H12I	108.2
H34A—C34—H34C	109.5	C122—C123—H12I	108.2
H34B—C34—H34C	109.5	H12H—C123—H12I	107.3
C36—C35—Sn2	108.8 (3)	C123—C124—H12J	109.5
C36—C35—H35A	109.9	C123—C124—H12K	109.5
Sn2—C35—H35A	109.9	H12J—C124—H12K	109.5
C36—C35—H35B	109.9	C123—C124—H12L	109.5
Sn2—C35—H35B	109.9	H12J—C124—H12L	109.5
H35A—C35—H35B	108.3	H12K—C124—H12L	109.5
C37—C36—C35	114.7 (4)	C126—C125—Sn5	114.0 (3)
C37—C36—H36A	108.6	C126—C125—H12M	108.7
C35—C36—H36A	108.6	Sn5—C125—H12M	108.7
C37—C36—H36B	108.6	C126—C125—H12N	108.7
C35—C36—H36B	108.6	Sn5—C125—H12N	108.7
H36A—C36—H36B	107.6	H12M—C125—H12N	107.6
C36—C37—C38	113.2 (4)	C125—C126—C127	113.4 (3)
C36—C37—H37A	108.9	C125—C126—H12O	108.9
C38—C37—H37A	108.9	C127—C126—H12O	108.9
C36—C37—H37B	108.9	C125—C126—H12P	108.9
C38—C37—H37B	108.9	C127—C126—H12P	108.9
H37A—C37—H37B	107.7	H12O—C126—H12P	107.7
C37—C38—H38A	109.5	C128—C127—C126	113.5 (3)
C37—C38—H38B	109.5	C128—C127—H12Q	108.9
H38A—C38—H38B	109.5	C126—C127—H12Q	108.9
C37—C38—H38C	109.5	C128—C127—H12R	108.9
H38A—C38—H38C	109.5	C126—C127—H12R	108.9
H38B—C38—H38C	109.5	H12Q—C127—H12R	107.7
N3—C39—S6	122.7 (3)	C127—C128—H12S	109.5
N3—C39—S5	119.1 (3)	C127—C128—H12T	109.5
S6—C39—S5	118.2 (2)	H12S—C128—H12T	109.5
N3—C40—C41	115.5 (3)	C127—C128—H12U	109.5
N3—C40—H40A	108.4	H12S—C128—H12U	109.5
C41—C40—H40A	108.4	H12T—C128—H12U	109.5
N3—C40—H40B	108.4	N9—C129—S18	122.8 (3)
C41—C40—H40B	108.4	N9—C129—S17	119.2 (3)
H40A—C40—H40B	107.5	S18—C129—S17	117.95 (19)
C46—C41—C42	118.5 (5)	N9—C130—C131	115.2 (3)
C46—C41—C40	124.3 (4)	N9—C130—H13B	108.5
C42—C41—C40	117.3 (4)	C131—C130—H13B	108.5
C43—C42—C41	120.2 (5)	N9—C130—H13C	108.5
C43—C42—H42	119.9	C131—C130—H13C	108.5
C41—C42—H42	119.9	H13B—C130—H13C	107.5
C44—C43—C42	120.9 (5)	C132—C131—C136	118.5 (4)
C44—C43—H43	119.5	C132—C131—C130	123.8 (4)
C42—C43—H43	119.5	C136—C131—C130	117.7 (4)
C43—C44—C45	119.9 (5)	C133—C132—C131	121.2 (4)
C43—C44—H44	120.1	C133—C132—H13D	119.4
C45—C44—H44	120.1	C131—C132—H13D	119.4
C44—C45—C46	119.8 (5)	C132—C133—C134	119.3 (5)
C44—C45—H45	120.1	C132—C133—H13E	120.3
C46—C45—H45	120.1	C134—C133—H13E	120.3
C41—C46—C45	120.7 (4)	C135—C134—C133	120.3 (5)
C41—C46—H46	119.7	C135—C134—H13F	119.9
C45—C46—H46	119.7	C133—C134—H13F	119.9
N3—C47—C48	111.6 (4)	C134—C135—C136	119.8 (5)
N3—C47—C49	112.5 (3)	C134—C135—H13G	120.1
C48—C47—C49	111.5 (3)	C136—C135—H13G	120.1
N3—C47—H47	107.0	C135—C136—C131	120.8 (4)
C48—C47—H47	107.0	C135—C136—H13H	119.6
C49—C47—H47	107.0	C131—C136—H13H	119.6
C47—C48—H48A	109.5	N9—C137—C139	110.3 (4)
C47—C48—H48B	109.5	N9—C137—C138	112.8 (4)
H48A—C48—H48B	109.5	C139—C137—C138	111.9 (4)
C47—C48—H48C	109.5	N9—C137—H13I	107.2
H48A—C48—H48C	109.5	C139—C137—H13I	107.2
H48B—C48—H48C	109.5	C138—C137—H13I	107.2
C47—C49—H49A	109.5	C137—C138—H13J	109.5
C47—C49—H49B	109.5	C137—C138—H13K	109.5
H49A—C49—H49B	109.5	H13J—C138—H13K	109.5
C47—C49—H49C	109.5	C137—C138—H13L	109.5
H49A—C49—H49C	109.5	H13J—C138—H13L	109.5
H49B—C49—H49C	109.5	H13K—C138—H13L	109.5
N4—C50—S8	122.0 (3)	C137—C139—H13M	109.5
N4—C50—S7	118.0 (3)	C137—C139—H13N	109.5
S8—C50—S7	120.0 (2)	H13M—C139—H13N	109.5
N4—C51—C52	113.4 (4)	C137—C139—H13O	109.5
N4—C51—H51A	108.9	H13M—C139—H13O	109.5
C52—C51—H51A	108.9	H13N—C139—H13O	109.5
N4—C51—H51B	108.9	N10—C140—S20	121.6 (3)
C52—C51—H51B	108.9	N10—C140—S19	118.4 (3)
H51A—C51—H51B	107.7	S20—C140—S19	120.0 (2)
C57—C52—C53	119.2 (4)	N10—C141—C142	115.0 (4)
C57—C52—C51	119.7 (4)	N10—C141—H14B	108.5
C53—C52—C51	121.1 (4)	C142—C141—H14B	108.5
C54—C53—C52	120.9 (4)	N10—C141—H14C	108.5
C54—C53—H53	119.6	C142—C141—H14C	108.5
C52—C53—H53	119.6	H14B—C141—H14C	107.5
C53—C54—C55	119.8 (5)	C143—C142—C147	119.0 (5)
C53—C54—H54	120.1	C143—C142—C141	121.5 (4)
C55—C54—H54	120.1	C147—C142—C141	119.5 (5)
C56—C55—C54	119.8 (4)	C144—C143—C142	120.8 (5)
C56—C55—H55	120.1	C144—C143—H14D	119.6
C54—C55—H55	120.1	C142—C143—H14D	119.6
C55—C56—C57	120.8 (5)	C143—C144—C145	119.4 (6)
C55—C56—H56	119.6	C143—C144—H14E	120.3
C57—C56—H56	119.6	C145—C144—H14E	120.3
C56—C57—C52	119.6 (5)	C146—C145—C144	120.5 (6)
C56—C57—H57	120.2	C146—C145—H14F	119.7
C52—C57—H57	120.2	C144—C145—H14F	119.7
C59—C58—N4	111.4 (4)	C145—C146—C147	119.9 (6)
C59—C58—C60	111.5 (4)	C145—C146—H14G	120.0
N4—C58—C60	112.6 (4)	C147—C146—H14G	120.0
C59—C58—H58	107.0	C146—C147—C142	120.4 (6)
N4—C58—H58	107.0	C146—C147—H14H	119.8
C60—C58—H58	107.0	C142—C147—H14H	119.8
C58—C59—H59A	109.5	N10—C148—C149	111.1 (4)
C58—C59—H59B	109.5	N10—C148—C150	112.0 (4)
H59A—C59—H59B	109.5	C149—C148—C150	111.5 (4)
C58—C59—H59C	109.5	N10—C148—H14I	107.3
H59A—C59—H59C	109.5	C149—C148—H14I	107.3
H59B—C59—H59C	109.5	C150—C148—H14I	107.3
C58—C60—H60A	109.5	C148—C149—H14J	109.5
C58—C60—H60B	109.5	C148—C149—H14K	109.5
H60A—C60—H60B	109.5	H14J—C149—H14K	109.5
C58—C60—H60C	109.5	C148—C149—H14L	109.5
H60A—C60—H60C	109.5	H14J—C149—H14L	109.5
H60B—C60—H60C	109.5	H14K—C149—H14L	109.5
C62—C61—Sn3	110.6 (3)	C148—C150—H15B	109.5
C62—C61—H61A	109.5	C148—C150—H15C	109.5
Sn3—C61—H61A	109.5	H15B—C150—H15C	109.5
C62—C61—H61B	109.5	C148—C150—H15D	109.5
Sn3—C61—H61B	109.5	H15B—C150—H15D	109.5
H61A—C61—H61B	108.1	H15C—C150—H15D	109.5
			
C1—Sn1—S1—C9	−73.37 (19)	Sn2—S8—C50—S7	−6.8 (2)
C5—Sn1—S1—C9	79.05 (19)	Sn2—S7—C50—N4	−173.3 (3)
S3—Sn1—S1—C9	−176.85 (14)	Sn2—S7—C50—S8	8.1 (3)
S2—Sn1—S1—C9	3.95 (14)	C50—N4—C51—C52	−95.9 (5)
S4—Sn1—S1—C9	−171.78 (14)	C58—N4—C51—C52	81.6 (5)
C1—Sn1—S2—C9	107.59 (18)	N4—C51—C52—C57	−121.3 (4)
C5—Sn1—S2—C9	−123.13 (19)	N4—C51—C52—C53	58.8 (5)
S1—Sn1—S2—C9	−4.14 (14)	C57—C52—C53—C54	−0.8 (7)
S3—Sn1—S2—C9	−5.53 (17)	C51—C52—C53—C54	179.1 (4)
S4—Sn1—S2—C9	170.66 (15)	C52—C53—C54—C55	0.3 (7)
C1—Sn1—S3—C20	71.11 (18)	C53—C54—C55—C56	0.7 (7)
C5—Sn1—S3—C20	−69.23 (17)	C54—C55—C56—C57	−1.3 (8)
S1—Sn1—S3—C20	177.28 (14)	C55—C56—C57—C52	0.9 (8)
S2—Sn1—S3—C20	178.57 (14)	C53—C52—C57—C56	0.1 (7)
S4—Sn1—S3—C20	0.64 (13)	C51—C52—C57—C56	−179.7 (4)
C1—Sn1—S4—C20	−113.77 (18)	C50—N4—C58—C59	95.1 (5)
C5—Sn1—S4—C20	113.72 (19)	C51—N4—C58—C59	−82.4 (5)
S1—Sn1—S4—C20	−6.23 (16)	C50—N4—C58—C60	−138.8 (4)
S3—Sn1—S4—C20	−0.66 (14)	C51—N4—C58—C60	43.7 (5)
S2—Sn1—S4—C20	−178.27 (14)	C65—Sn3—C61—C62	−8.5 (4)
C35—Sn2—S5—C39	71.52 (19)	S11—Sn3—C61—C62	−135.9 (3)
C31—Sn2—S5—C39	−79.40 (19)	S9—Sn3—C61—C62	137.4 (3)
S7—Sn2—S5—C39	175.57 (14)	S10—Sn3—C61—C62	75.3 (3)
S6—Sn2—S5—C39	−4.75 (13)	S12—Sn3—C61—C62	−77.2 (3)
S8—Sn2—S5—C39	169.74 (14)	Sn3—C61—C62—C63	−177.2 (3)
C35—Sn2—S6—C39	−107.42 (18)	C61—C62—C63—C64	173.4 (4)
C31—Sn2—S6—C39	123.70 (18)	C61—Sn3—C65—C66	135.0 (4)
S7—Sn2—S6—C39	5.50 (16)	S11—Sn3—C65—C66	−97.1 (4)
S5—Sn2—S6—C39	4.91 (14)	S9—Sn3—C65—C66	−9.0 (4)
S8—Sn2—S6—C39	−168.85 (14)	S10—Sn3—C65—C66	53.3 (4)
C35—Sn2—S7—C50	−73.90 (18)	S12—Sn3—C65—C66	−155.7 (4)
C31—Sn2—S7—C50	66.10 (18)	Sn3—C65—C66—C67	178.5 (4)
S5—Sn2—S7—C50	179.45 (14)	C65—C66—C67—C68	175.6 (5)
S6—Sn2—S7—C50	178.91 (14)	C70—N5—C69—S10	172.8 (3)
S8—Sn2—S7—C50	−4.32 (14)	C77—N5—C69—S10	−1.8 (6)
C35—Sn2—S8—C50	118.22 (18)	C70—N5—C69—S9	−6.3 (5)
C31—Sn2—S8—C50	−109.91 (19)	C77—N5—C69—S9	179.1 (3)
S7—Sn2—S8—C50	4.49 (14)	Sn3—S10—C69—N5	−177.8 (4)
S5—Sn2—S8—C50	10.93 (17)	Sn3—S10—C69—S9	1.3 (2)
S6—Sn2—S8—C50	−178.94 (15)	Sn3—S9—C69—N5	177.7 (3)
C61—Sn3—S9—C69	−73.52 (18)	Sn3—S9—C69—S10	−1.5 (2)
C65—Sn3—S9—C69	76.8 (2)	C69—N5—C70—C71	91.3 (5)
S11—Sn3—S9—C69	−179.32 (14)	C77—N5—C70—C71	−94.0 (5)
S10—Sn3—S9—C69	0.82 (14)	N5—C70—C71—C76	−6.5 (6)
S12—Sn3—S9—C69	−174.39 (14)	N5—C70—C71—C72	172.7 (4)
C61—Sn3—S10—C69	112.81 (17)	C76—C71—C72—C73	1.4 (7)
C65—Sn3—S10—C69	−119.68 (19)	C70—C71—C72—C73	−177.8 (4)
S11—Sn3—S10—C69	−1.11 (17)	C71—C72—C73—C74	1.0 (8)
S9—Sn3—S10—C69	−0.85 (14)	C72—C73—C74—C75	−2.1 (8)
S12—Sn3—S10—C69	173.90 (15)	C73—C74—C75—C76	0.9 (8)
C61—Sn3—S11—C80	71.68 (17)	C72—C71—C76—C75	−2.5 (7)
C65—Sn3—S11—C80	−67.03 (18)	C70—C71—C76—C75	176.6 (4)
S9—Sn3—S11—C80	178.83 (14)	C74—C75—C76—C71	1.4 (7)
S10—Sn3—S11—C80	179.07 (14)	C69—N5—C77—C78	−141.1 (4)
S12—Sn3—S11—C80	1.95 (14)	C70—N5—C77—C78	44.1 (6)
C61—Sn3—S12—C80	−117.07 (17)	C69—N5—C77—C79	93.2 (5)
C65—Sn3—S12—C80	112.1 (2)	C70—N5—C77—C79	−81.6 (5)
S11—Sn3—S12—C80	−2.06 (14)	C81—N6—C80—S12	−179.7 (3)
S9—Sn3—S12—C80	−7.49 (17)	C88—N6—C80—S12	3.4 (6)
S10—Sn3—S12—C80	−179.06 (14)	C81—N6—C80—S11	−1.0 (5)
C95—Sn4—S13—C99	−82.6 (2)	C88—N6—C80—S11	−177.9 (3)
C91—Sn4—S13—C99	75.43 (18)	Sn3—S12—C80—N6	−178.3 (4)
S15—Sn4—S13—C99	176.16 (15)	Sn3—S12—C80—S11	3.1 (2)
S14—Sn4—S13—C99	−1.58 (15)	Sn3—S11—C80—N6	177.7 (3)
S16—Sn4—S13—C99	171.58 (15)	Sn3—S11—C80—S12	−3.6 (3)
C95—Sn4—S14—C99	113.8 (2)	C80—N6—C81—C82	98.3 (5)
C91—Sn4—S14—C99	−109.15 (18)	C88—N6—C81—C82	−84.7 (5)
S15—Sn4—S14—C99	−2.68 (18)	N6—C81—C82—C87	−47.6 (5)
S13—Sn4—S14—C99	1.63 (15)	N6—C81—C82—C83	131.7 (4)
S16—Sn4—S14—C99	−171.43 (16)	C87—C82—C83—C84	−0.5 (7)
C95—Sn4—S15—C110	71.6 (2)	C81—C82—C83—C84	−179.9 (5)
C91—Sn4—S15—C110	−73.36 (19)	C82—C83—C84—C85	−1.3 (8)
S13—Sn4—S15—C110	−179.18 (16)	C83—C84—C85—C86	2.2 (8)
S14—Sn4—S15—C110	−175.20 (16)	C84—C85—C86—C87	−1.1 (8)
S16—Sn4—S15—C110	−2.00 (16)	C85—C86—C87—C82	−0.8 (7)
C95—Sn4—S16—C110	−107.1 (2)	C83—C82—C87—C86	1.6 (6)
C91—Sn4—S16—C110	111.66 (19)	C81—C82—C87—C86	−179.1 (4)
S15—Sn4—S16—C110	2.04 (16)	C80—N6—C88—C89	90.4 (5)
S13—Sn4—S16—C110	7.10 (19)	C81—N6—C88—C89	−86.7 (5)
S14—Sn4—S16—C110	175.58 (16)	C80—N6—C88—C90	−142.9 (5)
C121—Sn5—S17—C129	83.08 (19)	C81—N6—C88—C90	40.0 (6)
C125—Sn5—S17—C129	−76.53 (17)	C95—Sn4—C91—C92	10.2 (4)
S19—Sn5—S17—C129	−177.44 (13)	S15—Sn4—C91—C92	135.4 (3)
S18—Sn5—S17—C129	0.25 (13)	S13—Sn4—C91—C92	−138.8 (3)
S20—Sn5—S17—C129	−175.41 (13)	S14—Sn4—C91—C92	−75.3 (3)
C121—Sn5—S18—C129	−112.0 (2)	S16—Sn4—C91—C92	75.3 (3)
C125—Sn5—S18—C129	112.39 (16)	Sn4—C91—C92—C93	−177.8 (3)
S17—Sn5—S18—C129	−0.26 (13)	C91—C92—C93—C94	−175.2 (4)
S19—Sn5—S18—C129	4.07 (16)	C91—Sn4—C95—C96	−148.1 (8)
S20—Sn5—S18—C129	175.06 (14)	S15—Sn4—C95—C96	87.0 (8)
C121—Sn5—S19—C140	−66.83 (19)	S13—Sn4—C95—C96	0.2 (9)
C125—Sn5—S19—C140	75.87 (17)	S14—Sn4—C95—C96	−64.5 (8)
S17—Sn5—S19—C140	−176.42 (14)	S16—Sn4—C95—C96	147.7 (8)
S18—Sn5—S19—C140	179.58 (14)	C91—Sn4—C95—C96'	−127.6 (7)
S20—Sn5—S19—C140	4.87 (14)	S15—Sn4—C95—C96'	107.5 (7)
C121—Sn5—S20—C140	103.8 (2)	S13—Sn4—C95—C96'	20.7 (7)
C125—Sn5—S20—C140	−115.32 (17)	S14—Sn4—C95—C96'	−44.0 (7)
S17—Sn5—S20—C140	−7.27 (16)	S16—Sn4—C95—C96'	168.2 (7)
S19—Sn5—S20—C140	−5.02 (14)	C96'—C95—C96—C97	113 (2)
S18—Sn5—S20—C140	−179.72 (15)	Sn4—C95—C96—C97	173.1 (6)
C5—Sn1—C1—C2'	4.1 (5)	C95—C96—C97—C98	175.7 (10)
S1—Sn1—C1—C2'	151.4 (4)	C96—C95—C96'—C97'	−30.9 (12)
S3—Sn1—C1—C2'	−124.2 (4)	Sn4—C95—C96'—C97'	−161.4 (9)
S2—Sn1—C1—C2'	88.5 (4)	C95—C96'—C97'—C98'	−67.2 (15)
S4—Sn1—C1—C2'	−65.2 (4)	C100—N7—C99—S14	178.6 (3)
C5—Sn1—C1—C2	−14.4 (5)	C107—N7—C99—S14	−1.9 (6)
S1—Sn1—C1—C2	133.0 (4)	C100—N7—C99—S13	−5.5 (6)
S3—Sn1—C1—C2	−142.6 (4)	C107—N7—C99—S13	174.0 (3)
S2—Sn1—C1—C2	70.0 (4)	Sn4—S14—C99—N7	173.5 (4)
S4—Sn1—C1—C2	−83.7 (4)	Sn4—S14—C99—S13	−2.5 (2)
C2'—C1—C2—C3	34.8 (8)	Sn4—S13—C99—N7	−173.4 (3)
Sn1—C1—C2—C3	−174.5 (6)	Sn4—S13—C99—S14	2.7 (3)
C1—C2—C3—C4	177.9 (9)	C99—N7—C100—C101	−79.8 (5)
C2—C1—C2'—C3'	−36.6 (8)	C107—N7—C100—C101	100.7 (5)
Sn1—C1—C2'—C3'	−74.0 (8)	N7—C100—C101—C102	179.1 (4)
C1—C2'—C3'—C4'	−176.0 (9)	N7—C100—C101—C106	−0.8 (6)
C1—Sn1—C5—C6	144.7 (4)	C106—C101—C102—C103	0.0 (7)
S1—Sn1—C5—C6	−0.8 (4)	C100—C101—C102—C103	−179.8 (4)
S3—Sn1—C5—C6	−87.5 (4)	C101—C102—C103—C104	−0.7 (7)
S2—Sn1—C5—C6	61.0 (4)	C102—C103—C104—C105	−0.1 (8)
S4—Sn1—C5—C6	−146.0 (4)	C103—C104—C105—C106	1.5 (8)
Sn1—C5—C6—C7	179.6 (3)	C104—C105—C106—C101	−2.1 (7)
C5—C6—C7—C8	−178.3 (5)	C102—C101—C106—C105	1.4 (7)
C10—N1—C9—S2	−177.6 (3)	C100—C101—C106—C105	−178.8 (4)
C17—N1—C9—S2	1.6 (6)	C99—N7—C107—C108	101.5 (5)
C10—N1—C9—S1	3.9 (5)	C100—N7—C107—C108	−79.0 (5)
C17—N1—C9—S1	−177.0 (3)	C99—N7—C107—C109	−134.2 (5)
Sn1—S2—C9—N1	−172.4 (4)	C100—N7—C107—C109	45.4 (6)
Sn1—S2—C9—S1	6.2 (2)	C111—N8—C110—S16	173.2 (3)
Sn1—S1—C9—N1	171.6 (3)	C118—N8—C110—S16	−5.3 (6)
Sn1—S1—C9—S2	−7.0 (2)	C111—N8—C110—S15	−4.7 (6)
C9—N1—C10—C11	87.1 (5)	C118—N8—C110—S15	176.7 (3)
C17—N1—C10—C11	−92.0 (5)	Sn4—S16—C110—N8	178.9 (4)
N1—C10—C11—C16	−14.6 (6)	Sn4—S16—C110—S15	−3.1 (2)
N1—C10—C11—C12	166.8 (4)	Sn4—S15—C110—N8	−178.3 (3)
C16—C11—C12—C13	1.5 (7)	Sn4—S15—C110—S16	3.6 (3)
C10—C11—C12—C13	−179.9 (4)	C110—N8—C111—C112	−97.7 (5)
C11—C12—C13—C14	−0.5 (8)	C118—N8—C111—C112	80.9 (5)
C12—C13—C14—C15	−0.1 (8)	N8—C111—C112—C113	−122.4 (5)
C13—C14—C15—C16	−0.2 (8)	N8—C111—C112—C117	59.3 (6)
C12—C11—C16—C15	−1.8 (7)	C117—C112—C113—C114	1.2 (8)
C10—C11—C16—C15	179.6 (4)	C111—C112—C113—C114	−177.2 (5)
C14—C15—C16—C11	1.2 (7)	C112—C113—C114—C115	−0.1 (10)
C9—N1—C17—C19	−93.1 (5)	C113—C114—C115—C116	−0.8 (9)
C10—N1—C17—C19	86.0 (5)	C114—C115—C116—C117	0.6 (8)
C9—N1—C17—C18	141.9 (4)	C115—C116—C117—C112	0.5 (7)
C10—N1—C17—C18	−38.9 (5)	C113—C112—C117—C116	−1.4 (7)
C21—N2—C20—S4	179.3 (3)	C111—C112—C117—C116	177.0 (4)
C28—N2—C20—S4	3.6 (5)	C110—N8—C118—C119	95.9 (6)
C21—N2—C20—S3	−1.1 (5)	C111—N8—C118—C119	−82.7 (6)
C28—N2—C20—S3	−176.9 (3)	C110—N8—C118—C120	−139.8 (5)
Sn1—S4—C20—N2	−179.5 (3)	C111—N8—C118—C120	41.6 (6)
Sn1—S4—C20—S3	1.0 (2)	C125—Sn5—C121—C122	115.7 (4)
Sn1—S3—C20—N2	179.3 (3)	S17—Sn5—C121—C122	−36.2 (5)
Sn1—S3—C20—S4	−1.2 (2)	S19—Sn5—C121—C122	−121.9 (4)
C20—N2—C21—C22	98.0 (4)	S18—Sn5—C121—C122	29.1 (4)
C28—N2—C21—C22	−86.1 (4)	S20—Sn5—C121—C122	178.3 (4)
N2—C21—C22—C27	133.3 (4)	Sn5—C121—C122—C123	172.0 (4)
N2—C21—C22—C23	−46.4 (5)	C121—C122—C123—C124	172.3 (5)
C27—C22—C23—C24	0.8 (6)	C121—Sn5—C125—C126	−13.6 (4)
C21—C22—C23—C24	−179.5 (4)	S17—Sn5—C125—C126	138.6 (3)
C22—C23—C24—C25	−0.3 (6)	S19—Sn5—C125—C126	−135.6 (3)
C23—C24—C25—C26	−1.2 (7)	S18—Sn5—C125—C126	75.3 (3)
C24—C25—C26—C27	2.2 (7)	S20—Sn5—C125—C126	−76.2 (3)
C25—C26—C27—C22	−1.7 (7)	Sn5—C125—C126—C127	176.8 (3)
C23—C22—C27—C26	0.2 (6)	C125—C126—C127—C128	173.0 (4)
C21—C22—C27—C26	−179.5 (4)	C130—N9—C129—S18	172.9 (3)
C20—N2—C28—C30	94.9 (5)	C137—N9—C129—S18	−0.9 (5)
C21—N2—C28—C30	−81.1 (5)	C130—N9—C129—S17	−6.2 (5)
C20—N2—C28—C29	−138.1 (5)	C137—N9—C129—S17	−179.9 (3)
C21—N2—C28—C29	45.9 (6)	Sn5—S18—C129—N9	−178.7 (3)
C35—Sn2—C31—C32	−150.6 (3)	Sn5—S18—C129—S17	0.4 (2)
S7—Sn2—C31—C32	81.4 (3)	Sn5—S17—C129—N9	178.7 (3)
S5—Sn2—C31—C32	−6.7 (4)	Sn5—S17—C129—S18	−0.4 (2)
S6—Sn2—C31—C32	−68.5 (3)	C129—N9—C130—C131	89.8 (5)
S8—Sn2—C31—C32	140.1 (4)	C137—N9—C130—C131	−96.3 (4)
Sn2—C31—C32—C33	177.5 (3)	N9—C130—C131—C132	−2.4 (6)
C31—C32—C33—C34	175.0 (5)	N9—C130—C131—C136	177.2 (3)
C31—Sn2—C35—C36	10.0 (4)	C136—C131—C132—C133	−1.8 (7)
S7—Sn2—C35—C36	138.4 (3)	C130—C131—C132—C133	177.9 (4)
S5—Sn2—C35—C36	−135.8 (3)	C131—C132—C133—C134	1.3 (7)
S6—Sn2—C35—C36	−72.9 (3)	C132—C133—C134—C135	0.6 (7)
S8—Sn2—C35—C36	79.9 (3)	C133—C134—C135—C136	−1.8 (7)
Sn2—C35—C36—C37	178.2 (4)	C134—C135—C136—C131	1.3 (7)
C35—C36—C37—C38	−171.6 (5)	C132—C131—C136—C135	0.5 (6)
C40—N3—C39—S6	176.9 (3)	C130—C131—C136—C135	−179.2 (4)
C47—N3—C39—S6	−3.2 (5)	C129—N9—C137—C139	89.8 (5)
C40—N3—C39—S5	−4.0 (5)	C130—N9—C137—C139	−84.2 (5)
C47—N3—C39—S5	175.9 (3)	C129—N9—C137—C138	−144.2 (4)
Sn2—S6—C39—N3	171.7 (4)	C130—N9—C137—C138	41.8 (5)
Sn2—S6—C39—S5	−7.4 (2)	C141—N10—C140—S20	−172.6 (3)
Sn2—S5—C39—N3	−170.8 (3)	C148—N10—C140—S20	7.0 (6)
Sn2—S5—C39—S6	8.3 (2)	C141—N10—C140—S19	4.9 (6)
C39—N3—C40—C41	−81.4 (5)	C148—N10—C140—S19	−175.5 (3)
C47—N3—C40—C41	98.8 (4)	Sn5—S20—C140—N10	−174.9 (4)
N3—C40—C41—C46	3.8 (6)	Sn5—S20—C140—S19	7.7 (2)
N3—C40—C41—C42	−175.9 (4)	Sn5—S19—C140—N10	173.4 (3)
C46—C41—C42—C43	0.8 (7)	Sn5—S19—C140—S20	−9.0 (3)
C40—C41—C42—C43	−179.5 (4)	C140—N10—C141—C142	96.5 (5)
C41—C42—C43—C44	−1.7 (8)	C148—N10—C141—C142	−83.2 (5)
C42—C43—C44—C45	1.5 (8)	N10—C141—C142—C143	−58.8 (6)
C43—C44—C45—C46	−0.5 (8)	N10—C141—C142—C147	124.4 (5)
C42—C41—C46—C45	0.2 (7)	C147—C142—C143—C144	0.9 (7)
C40—C41—C46—C45	−179.5 (4)	C141—C142—C143—C144	−175.9 (4)
C44—C45—C46—C41	−0.4 (8)	C142—C143—C144—C145	−0.9 (8)
C39—N3—C47—C48	94.4 (5)	C143—C144—C145—C146	−0.2 (9)
C40—N3—C47—C48	−85.8 (4)	C144—C145—C146—C147	1.4 (11)
C39—N3—C47—C49	−139.4 (4)	C145—C146—C147—C142	−1.4 (10)
C40—N3—C47—C49	40.4 (5)	C143—C142—C147—C146	0.2 (8)
C51—N4—C50—S8	170.1 (3)	C141—C142—C147—C146	177.1 (5)
C58—N4—C50—S8	−7.3 (6)	C140—N10—C148—C149	−98.4 (5)
C51—N4—C50—S7	−8.5 (5)	C141—N10—C148—C149	81.2 (5)
C58—N4—C50—S7	174.1 (3)	C140—N10—C148—C150	136.1 (4)
Sn2—S8—C50—N4	174.7 (4)	C141—N10—C148—C150	−44.2 (6)
